# Enhanced HDL Functionality in Small HDL Species Produced Upon Remodeling of HDL by Reconstituted HDL, CSL112: Effects on Cholesterol Efflux, Anti-Inflammatory and Antioxidative Activity

**DOI:** 10.1161/CIRCRESAHA.116.308685

**Published:** 2016-09-01

**Authors:** Svetlana A. Didichenko, Alexei V. Navdaev, Alexandre M.O. Cukier, Andreas Gille, Patrick Schuetz, Martin O. Spycher, Patrice Thérond, M. John Chapman, Anatol Kontush, Samuel D. Wright

**Affiliations:** From the CSL Behring AG, Berne, Switzerland (S.A.D., A.V.N., P.S., M.O.S.); National Institute for Health and Medical Research (INSERM), UMR-ICAN 1166, Paris, France (A.M.O.C., M.J.C., A.K.); University of Pierre and Marie Curie - Paris 6, France (A.M.O.C., M.J.C., A.K.); Pitié – Salpétrière University Hospital; ICAN, Paris, France (A.M.O.C., M.J.C., A.K.); CSL Limited, Parkville, VIC, Australia (A.G.); AP-HP, HUPS Hôpital de Bicêtre, Le Kremlin-Bicêtre, France (P.T.); and CSL Behring, King of Prussia, PA (S.D.W.).

**Keywords:** antioxidant, apolipoprotein A-I, cholesterol, high-density lipoproteins, inflammation

## Abstract

**Rationale:**

CSL112, human apolipoprotein A-I (apoA-I) reconstituted with phosphatidylcholine, is known to cause a dramatic rise in small high-density lipoprotein (HDL).

**Objective:**

To explore the mechanisms by which the formation of small HDL particles is induced by CSL112.

**Methods and Results:**

Infusion of CSL112 into humans caused elevation of 2 small diameter HDL fractions and 1 large diameter fraction. Ex vivo studies showed that this remodeling does not depend on lipid transfer proteins or lipases. Rather, interaction of CSL112 with purified HDL spontaneously gave rise to 3 HDL species: a large, spherical species composed of apoA-I from native HDL and CSL112; a small, disc-shaped species composed of apoA-I from CSL112, but smaller because of the loss of phospholipids; and the smallest species, lipid-poor apoA-I composed of apoA-I from HDL and CSL112. Time-course studies suggest that remodeling occurs by an initial fusion of CSL112 with HDL and subsequent fission leading to the smaller forms. Functional studies showed that ATP-binding cassette transporter 1–dependent cholesterol efflux and anti-inflammatory effects in whole blood were carried by the 2 small species with little activity in the large species. In contrast, the ability to inactivate lipid hydroperoxides in oxidized low-density lipoprotein was carried predominantly by the 2 largest species and was low in lipid-poor apoA-I.

**Conclusions:**

We have described a mechanism for the formation of small, highly functional HDL species involving spontaneous fusion of discoidal HDL with spherical HDL and subsequent fission. Similar remodeling is likely to occur during the life cycle of apoA-I in vivo.

## Abstract 2

Supplemental Digital Content is available in the text.

High-density lipoprotein (HDL) cholesterol (HDL-C) levels poorly predict the ability of plasma to promote cholesterol efflux from cells and to reduce inflammatory responses of cells ex vivo. For example, plasma samples from patients with median levels of HDL-C may have cholesterol efflux values either well above or below the median.^1^ Importantly, efflux values to apolipoprotein B–depleted plasma are much stronger predictors of risk of coronary heart disease than HDL-C values.^2–4^ This illustrates both the disconnection of functional measures of HDL-C and the potential value of functional measures in understanding human disease. This point is further emphasized by observations on the cholesterylester transfer protein (CETP) inhibitor, dalcetrapib. Treatment with dalcetrapib elevated HDL-C by ≈30% but increased total cholesterol efflux (primarily ATP-binding cassette transporter [ABCA1]–independent efflux) by <10%.^5^

**Editorial, see p 704**

Recent work has described a mechanism by which measures of HDL-C and cholesterol efflux may diverge. The largest source of patient-to-patient variation in cholesterol efflux is the ABCA1-dependent fraction of efflux.^1^ The ABCA1 transporter efficiently moves cellular cholesterol exclusively to small, lipid-poor, protein-rich HDL species but is practically inactive with large, cholesterol-rich HDL species.^6^ In keeping with these findings, ABCA1-dependent efflux capacity in human plasma samples correlates closely with levels of the smallest HDL form,^7^ which is known as preβ1-HDL, very small-HDL (VS-HDL),^8^ or lipid-poor apolipoprotein A-I (apoA-I). Thus, the HDL species that make the largest contribution to cholesterol efflux carry the least cholesterol. The factors that regulate formation of these highly functional but low-cholesterol species of HDL are key to understanding the relationship of HDL to cardiovascular risk. About 5% of plasma apoA-I is present as a lipid-poor species as measured with 2-dimensional (2D) gel electrophoresis^9^ or with a specific ELISA.^10^

The steps by which lipid-poor apoA-I acquires cholesterol and grows from discs to spheres through the action of lecithin:cholesterol acyltransferase (LCAT) are relatively well understood.^11,12^ In contrast, the process by which apoA-I is recycled from mature HDL to become lipid-poor is less well studied. One proposed process involves lipolysis of chylomicrons and generation of lipid-poor apoA-I.^13^ In a similar fashion, lipolysis of HDL by hepatic and endothelial lipase can lead to particle remodeling with the release of lipid-poor apoA-I.^14,15^ In another proposed process, CETP catalyzes interaction of HDL with triglyceride-rich lipoproteins to yield lipid-poor apoA-I and HDL of a larger size,^16^ whereas phospholipid transfer protein (PLTP) may catalyze interaction of HDL particles to yield both lipid-poor apoA-I and lipoproteins of a larger size.^17,18^ A common feature of all these processes is that all are catalyzed by specific lipid transfer proteins or lipases.

We have studied formation of lipid-poor apoA-I in blood and plasma with a novel agent. CSL112 is human apoA-I reconstituted into disc-shaped lipoproteins with phosphatidylcholine in a form suitable for intravenous infusion.^19^ CSL112 is currently in phase II clinical development for the treatment of acute coronary syndrome. We have previously observed that infusion of CSL112 into healthy volunteers causes HDL remodeling with an extraordinary rise in lipid-poor apoA-I measured by ELISA with levels rising ≤36-fold.^20^ Here we describe the mechanism behind this elevation and characterize the composition and biological activities of the products of HDL remodeling. To our surprise, we found that generation of lipid-poor apoA-I seems not to require triglyceride-rich lipoproteins, CETP, PLTP, or lipases. Rather, the lipid-poor apoA-I is formed on the spontaneous interaction of CSL112 with HDL2 or HDL3, deriving equally from the mature HDL and from CSL112. We found that the small HDL species resulting from this remodeling mediate the majority of the ABCA1-dependent cholesterol efflux and exert potent anti-inflammatory effects.

## Methods

An expanded Methods section is available in Material section Online Data Supplement.

### Reconstituted HDL

CSL112 particles contain 2 molecules of apoA-I and 110 molecules of phosphatidylcholine per particle as described.^19^ HDL labeling, lipoprotein isolation, and in vitro incubations are described in the Online Data Supplement.

### Electron Microscopy

Negative stain electron microscopy was performed as described previously.^21^

### Cholesterol Efflux Assay

The capacity of the HDL to efflux cholesterol was assessed using [^3^H]cholesterol-loaded RAW264.7 macrophages as previously described.^19^

### Work With Human Subjects

Plasma samples of the healthy subjects from a completed phase I clinical trial (NCT01281774)^22^ were obtained at multiple time points for assessment of apoA-I by nephelometry (Pacific Biomarkers Inc) and 2D-gel (3%–35% concave gradient) electrophoresis (Boston Heart Diagnostics).

Western blotting of 2D-gradient gels was conducted using polyclonal anti–apoA-I and diluted anti-albumin antibodies. Relative apoA-I content in HDL subfractions on blots was quantified by densitometry as a percentage of the total combined signal (100% per blot). Absolute HDL subfraction concentrations were then calculated as the measured percentage of the apoA-I concentrations for matching time points.

### Stimulation of Peripheral Blood Mononuclear Cells, Cytokine Measurements, and Cell Lysis

Peripheral blood mononuclear cells (PBMCs) were isolated from buffy coats by density gradient centrifugation using Ficoll-Pague Plus (GE Healthcare). PBMCs were cultured in RPMI 1640 supplemented with 5% (v/v) fetal calf serum (Gibco), 2 mmol/L l-glutamine, 100 U/mL penicillin, and 100 μg/mL streptomycin at a density of 1 to 1.5×10^6^ cells per well in 48-well plates. Cells were stimulated with 1 µg/mL phytohemagglutinin M (Calbiochem) in the presence or absence of different HDL preparations and incubated for 20 hours at 37°C in a CO_2_ incubator. To prevent potential effects of endotoxin contamination, cell culture medium was supplemented with 1 μg/mL polymixin B (Sigma-Aldrich); HDL and phytohemagglutinin M were preincubated with polymyxin B for 15 minutes before being added to PBMC. Tumor necrosis factor-α, interleukin-1β (IL-1β), IL-6, and macrophage inflammatory protein-1β were measured in cell-free supernatants using Human Cytokine Magnetic 4-Plex Panel (R&D Systems).

### Phospholipid Hydroperoxide Inactivating Capacity of HDL

Reference low-density lipoprotein (LDL) obtained from a healthy normolipidemic subject was preoxidized at 40-mg total cholesterol/dL with 4-mmol/L 2,2′-Azobis(2-amidinopropane) dihydrochloride for 6 hours at 37°C. Oxidative modification was terminated by addition of EDTA (10 µmol/L) and butylated hydroxytoluene (10 µmol/L). Oxidized LDL (oxLDL) was dialyzed against PBS at 4°C to remove EDTA and excess butylated hydroxytoluene, and incubated at a concentration of 20 mg total cholesterol/dL for 4 hours in the presence or absence of HDL (8 mg total protein/dL) in PBS at 37°C. EDTA (100 µmol/L) was present to inhibit lipid peroxidation during the incubation. Phospholipid hydroperoxides (PLOOH) were quantified in the reaction mixture before and after incubation by high-performance liquid chromatography with chemiluminescent detection as described elsewhere.^23^ To measure apoA-I content of native and oxidized methionine (Met) residues, HDL was subjected to high-performance liquid chromatography with ultraviolet detection at 214 nm as previously described.^23^

### Statistics

Values are presented as mean±SDs. All results were analyzed for statistical significance using 1-way ANOVA followed by Dunnet post hoc test. Statistical significance was set at *P*<0.05.

## Results

### CSL112 Is Rapidly Remodeled on Infusion into Human Subjects

**Figure 1.**
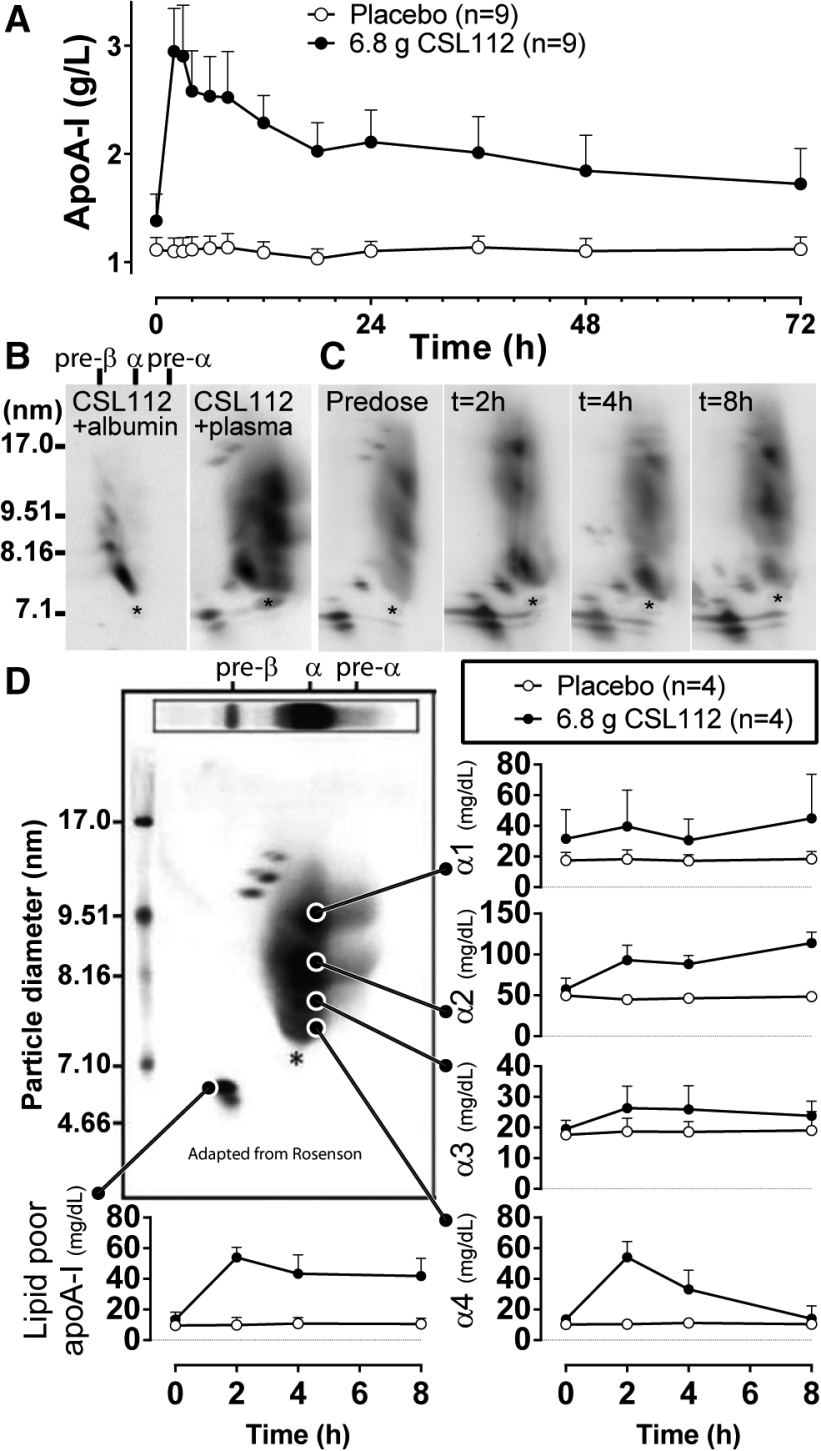
**Infusion with CSL112 leads to changes in concentration and size distribution of plasma high-density lipoprotein (HDL) subclasses. A**, Apolipoprotein A-I (ApoA-I) pharmacokinetic profile (mean±SD) after the infusion of 6.8 g (n=9) CSL112 or placebo (n=9). **B**, Electrophoretic mobility of CSL112 on 2-dimensional (2D) gel. **Left**, CSL112 migrates as ≈7.6 nm particle with slow-α mobility relative to albumin. Human serum albumin was premixed with CSL112 to allow orientation of HDL subpopulation relative to the albumin immunoreactivity (marked by asterisk). **Right**, Mobility of CSL112 relative to plasma HDL. CSL112 (1 mg/mL) added to plasma at 0°C to minimize remodeling has slow-α mobility as assessed by 2D-gel and is distinct from endogenous HDL population. **C**, Dynamics of plasma HDL remodeling induced by CSL112. Shown are representative 2D-gels of a female 21-year-old subject receiving 6.8 g CSL112. **D**, Time course changes in the abundance of αHDL subpopulation and lipid-poor apoA-I (preβ1-HDL).The concentration of individual subfractions were calculated from relative abundance of the subfraction and apoA-I concentration in the sample (n=4 per group). For orientation, shown is a prototypical 2D-gel image adapted from Rosenson et al^8^ indicating major HDL-subpopulation (adapted with permission of the publisher. Copyright © 2011, American Association for Clinical Chemistry Inc).

HDL remodeling was studied in selected healthy subjects receiving infusions of 6.8 g of CSL112 or placebo (n=4 per group) from a completed phase I clinical trial (NCT01281774).^22^ Infusion with CSL112 resulted in immediate elevation of plasma apoA-I (Figure 1A) and profound changes in concentration and size distribution of plasma HDL subclasses (Figure 1C) as assessed by 2D-gel electrophoresis and Western blotting with anti–apoA-I antibody. Individual HDL subpopulation, α-migrating (α1, α2, α3, and α4 and preβ-migrating (lipid-poor apoA-I) HDL particles were identified,^24^ and their concentrations were quantified by densitometry as described in Materials and Methods section of this article (Figure 1D). Figure 1B and 1C demonstrate that at 2- to 4-hour postinfusion parent CSL112 was no longer seen in plasma, and several HDL species became elevated: lipid-poor apoA-I (preβ1-HDL), small (α3 and α4) HDL, and large (α1 and α2) HDL. The most prominent increase in concentration was observed for lipid-poor apoA-I and α4 HDL (Figure 1D). These data suggest that CSL112 caused HDL particle remodeling and was incorporated into the endogenous HDL pool. CSL112 does not fuse with large apolipoprotein B (apoB)–containing lipoproteins or affect the distribution of apolipoprotein E (apoE; Online Figure I). Concentrations of apoA-II did not change immediately after the infusion of CSL112. However at 24 hours post infusion, there was a modest dose-dependent increase in apoA-II not exceeding 10% of the baseline apoA-II concentration (Online Figure II).

### HDL Remodeling Induced by CSL112 in Plasma Ex Vivo Is a Time- and Temperature-Dependent Process

The early effects of the infusion of CSL112 into human subjects, including a rapid increase in concentration of lipid-poor apoA-I, apparent changes in the HDL2 and HDL3 particle size distribution and disappearance of parent CSL112 (Figure 1B and 1C; Online Figure III), were nearly identical to those observed on short-term incubation of CSL112 with human plasma ex vivo.^19^ Additional studies, therefore, used ex vivo incubations to identify factors underlying these changes. First, we examined whether inhibition or stimulation of the plasma factors LCAT, CETP, PLTP, and secretory phospholipase 2 could affect the observed formation of lipid-poor apoA-I after the addition of CSL112 to human plasma. Incubations were carried out for 1 hour at 37°C in the presence of LCAT inhibitor (5,5-dithiobis(2-nitrobenzoic acid), a CETP inhibitor (Torcetrapib), inhibitors of secretory phospholipase 2 (LY 311727) or a PLTP stimulator (4-(2-aminoethyl)-benzenesulphonyl fluoride). We observed no significant differences in levels of lipid-poor apoA-I (measured by ELISA) and in the size distribution of HDL population after incubation of CSL112 with control plasma or plasma treated with different inhibitors (Online Figures IVA and IVB). Importantly, addition of CSL112 to plasma dramatically elevated both ABCA1-dependent and ABCA1-independent efflux, and this elevation was similar under all conditions tested (Online Figure IVC). Additional confirmatory studies showed normal remodeling in plasma from mice deficient in PLTP or LCAT (Online Figure V).

**Figure 2.**
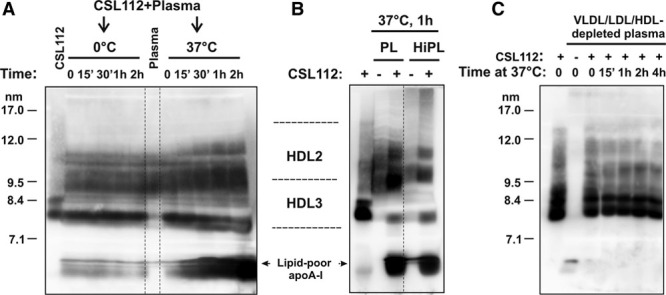
**High-density lipoprotein (HDL) remodeling induced by CSL112 in plasma ex vivo is time and temperature dependent.** Heat-sensitive plasma factors are not required. Plasma samples were subjected to nondenaturing polyacrylamide gradient gel electrophoresis followed by Western blotting with an anti–apolipoprotein A-I (apoA-I) antibody. CSL112 was included in as a control. The positions of migration of native HDL3 and HDL2 subpopulation and lipid-poor apoA-I are indicated. **A**, CSL112 was incubated with normolipidemic human plasma at a final concentration of 1-mg protein/mL for the indicated periods of time at 0°C or 37°C. **B**, HDL remodeling is not affected by heat inactivation of the plasma. CSL112 was added to normal plasma (PL) or heat-inactivated plasma (HiPL, heat treatment for 1 hour at 56°C) for 1 hour at 37°C. **C**, Human plasma was depleted of very low-density lipoprotein (VLDL)/LDL and HDL by ultracentrifugation at a density of 1.25 g/mL for 24 h. The bottom fraction representing lipoprotein-depleted plasma was incubated with CSL112 for the indicated periods of time at 37°C.

To evaluate the temperature dependence of HDL remodeling, CSL112 was incubated with plasma for different periods of time at 0°C or at 37°C, and plasma lipoproteins were separated on nondenaturing gradient gels followed by the detection of apoA-I–containing particles by Western blotting using an anti–apoA-I antibody. Incubation at 37°C led to changes in HDL particle size distribution and progressive accumulation of lipid-poor apoA-I with clear effects already visible by 15 to 30 minutes, whereas incubation at 0°C resulted in no remodeling (Figure 2A). Heating of the plasma for 1 hour at 56°C before incubation with CSL112 for another hour did not inhibit the HDL remodeling suggesting that heat-sensitive factors present in normal plasma are not required (Figure 2B). However, when lipoproteins (very low-density lipoprotein/LDL and HDL) were depleted from normal plasma by density gradient centrifugation, even prolonged incubation with CSL112 for 4 hours at 37°C led to no increase in levels of lipid-poor apoA-I (Figure 2C). Moreover, trace amount of lipid-poor apoA-I present in very low-density lipoprotein/LDL and HDL-depleted plasma appeared to be fully incorporated into CSL112 particles during incubation. These data suggest that a direct interaction between CSL112 and plasma lipoproteins is necessary for remodeling in the plasma compartment.

### Interaction Between HDL and CSL112 Is Sufficient for HDL Particle Remodeling

**Figure 3.**
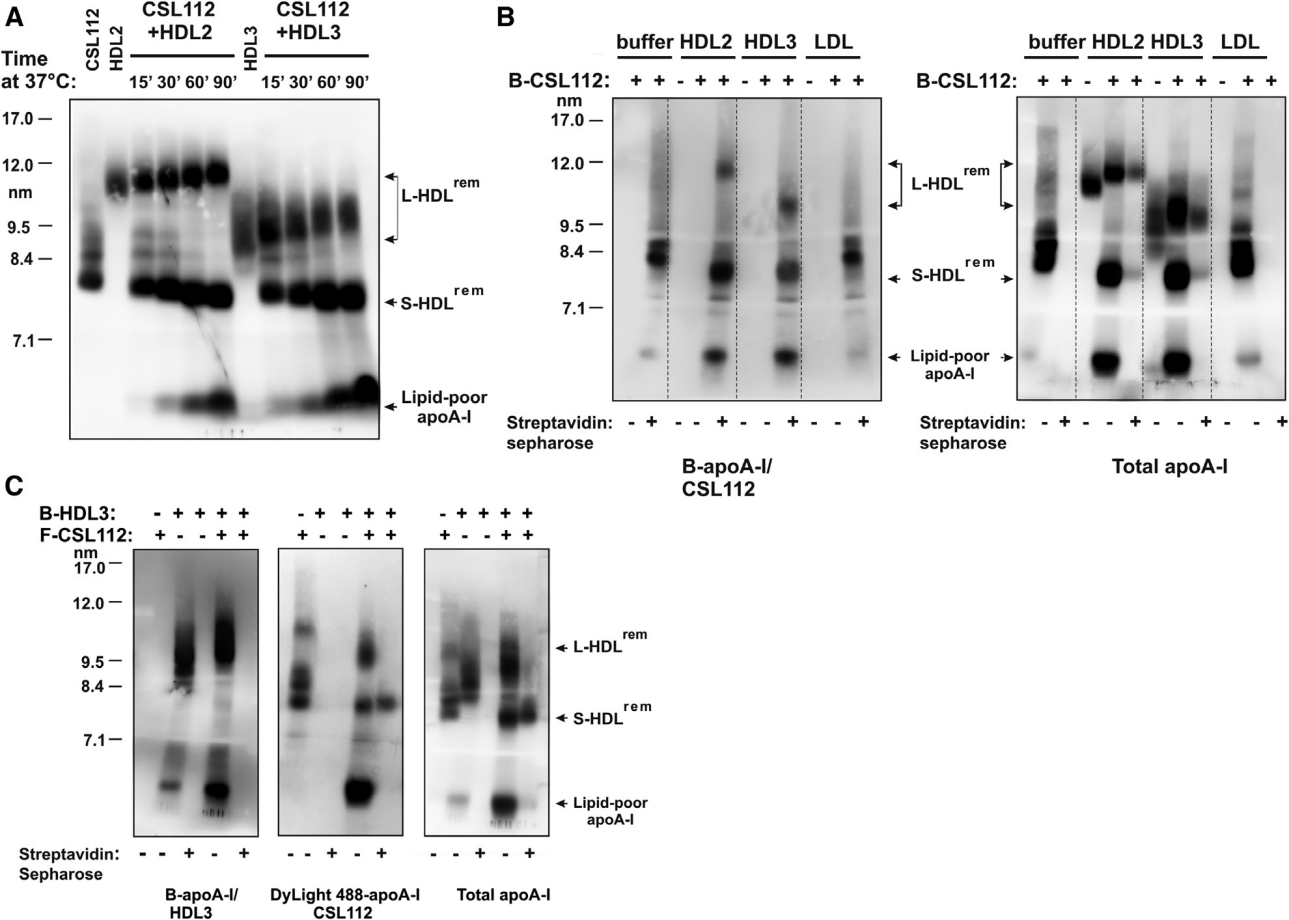
**Interaction between high-density lipoprotein (HDL) and CSL112 is sufficient for the particle remodeling in vitro. Products of remodeling contain apolipoprotein A-I (apoA-I) derived from both HDL and CSL112. A**, CSL112 promotes particle remodeling of different HDL subclasses in vitro. CSL112 was incubated with purified HDL3 or HDL2 for the indicated periods of time at 37°C. Lipoprotein mixtures were subjected to nondenaturing polyacrylamide gradient gel electrophoresis and analyzed by Western blotting with an anti-apoA-I antibody. The position of migration of remodeled HDL subspecies, L-HDL^rem^ and S-HDL^rem^, and lipid-poor apoA-I is indicated. **B**, ApoA-I derived from CSL112 is found in large HDL subspecies and lipid-poor apoA-I. Biotinylated-CSL112 (B-CSL112) was incubated with purified HDL2, HDL3, or LDL for 1 h at 37°C. In controls, each lipoprotein was incubated in buffer alone. Where indicated, the reaction mixtures were treated with streptavidin sepharose beads to remove lipoproteins containing biotinylated apoA-I. Western blotting was performed with NeutrAvidin to detect biotinylated apoA-I (B-apoA-I/CSL112, **left**) and with an anti-apoA-I antibody (total apoA-I, **right**). **C**, Lipid-poor apoA-I contains apoA-I from both CSL112 and HDL. Fluorescent CSL112 (F-CSL112) containing apoA-I labeled with DyLight488 was incubated with biotinylated-HDL3 (B-HDL3) for 1 h at 37°C. Samples were treated with streptavidin sepharose beads and analyzed for total apoA-I content (**right**) and biotinylated apoA-I content (B-apoA-I/HDL3, **left**) as described above. Fluorescent apoA-I (DyLight488-apoA-I/CSL112) was visualized by fluorescence imaging of the blots (**middle**). The positions of migration of L-HDL^rem^, S-HDL^rem^, and lipid-poor apoA-I are indicated.

To determine whether interaction with HDL alone is sufficient for particle remodeling by CSL112, we used purified HDL2 or HDL3 for the incubation with CSL112 at 37°C for various periods of time and assessed HDL particle size by nondenaturing polyacrylamide gradient gel electrophoresis followed by Western blotting with anti–apoA-I antibody. In this reconstituted system, HDL is progressively converted into larger particles (L-HDL^rem^, ≈10–12 nm; Figure 3A). Simultaneously, large amount of lipid-poor apoA-I (≈5.4 nm) are produced. Comparable changes were seen with either HDL3 or HDL2. These changes were accompanied by simultaneous, progressive reduction in the size of parent CSL112 particles (≈7.6–7.9) and accumulation of smaller species (S-HDL^rem^, ≈7.3–7.6 nm). In contrast, incubation of CSL112, HDL2, or HDL3 by themselves lead to no changes in size distribution or amount of lipid-poor apoA-I (Online Figure VI). Thus, addition of CSL112 induces remodeling of purified HDL and the changes seen after the admixture of these purified species recapitulated the remodeling in whole plasma.

### Origin and Destination of apoA-I During Remodeling

Protein labeling of CSL112 or HDL with biotin or with DyLight 488 fluorophore was undertaken to follow the distribution of specifically labeled apoA-I among the 3 products of particle remodeling. When biotinylated CSL112 was incubated with purified LDL for 1 hour at 37°C, we observed no remodeling of biotinylated CSL112 particles and no binding of biotinylated apoA-I to LDL (Figure 3B). However, after the incubation of biotinylated CSL112 with HDL2 or HDL3, all 3 products of remodeling (L-HDL^rem^, S-HDL^rem^, and lipid-poor apoA-I) were found to incorporate biotin label and could be removed from incubation mixtures with streptavidin sepharose (Figure 3B, right). These data suggest that CSL112-derived apoA-I moves to all species during remodeling.

Consistently, on incubation of CSL112 labeled with DyLight 488 fluorophore and biotinylated HDL3, fluorescently labeled apoA-I derived from CSL112 was also detected in all products of the remodeling (Figure 3C, middle). In contrast, placement of the biotin label on HDL3 showed that HDL3-derived biotinylated apoA-I was primarily found in L-HDL^rem^ and lipid-poor apoA-I, but not in S-HDL^rem^ (Figure 3C, left). Precipitation with streptavidin beads depleted fluorescently labeled L-HDL^rem^ and lipid-poor apoA-I from the incubation mixtures (Figure 3C, middle) but did not deplete S-HDL^rem^.

These findings indicate that during remodeling apoA-I moves from HDL3 to L-HDL^rem^ and to the lipid-poor apoA-I fraction but not to S-HDL^rem^. The ability of streptavidin to precipitate both biotinylated and fluorescently labeled apoA-I in L-HDL^rem^ indicates that apoA-I originating from HDL3 and CSL112 comes to reside on the same particle. We further presume that under the conditions of our experiments, lipid-poor apoA-I reaches a concentration sufficient to drive the well-characterized, concentration-dependent self-association of apoA-I to form dimers and multimers,^25^ and this oligomerization accounts for the coprecipitation.

The movement of apoA-I from CSL112 and HDL3 to the 3 products of remodeling is consistent with a model involving transient fusion of CSL112 with HDL3 and subsequent fission to lead to the products (Online Figure VII). The predictions of such a model were tested in time course studies and by following the movement of lipid during remodeling.

### Dynamics of Transfer of Protein and Lipid Content During Particle Remodeling

**Figure 4.**
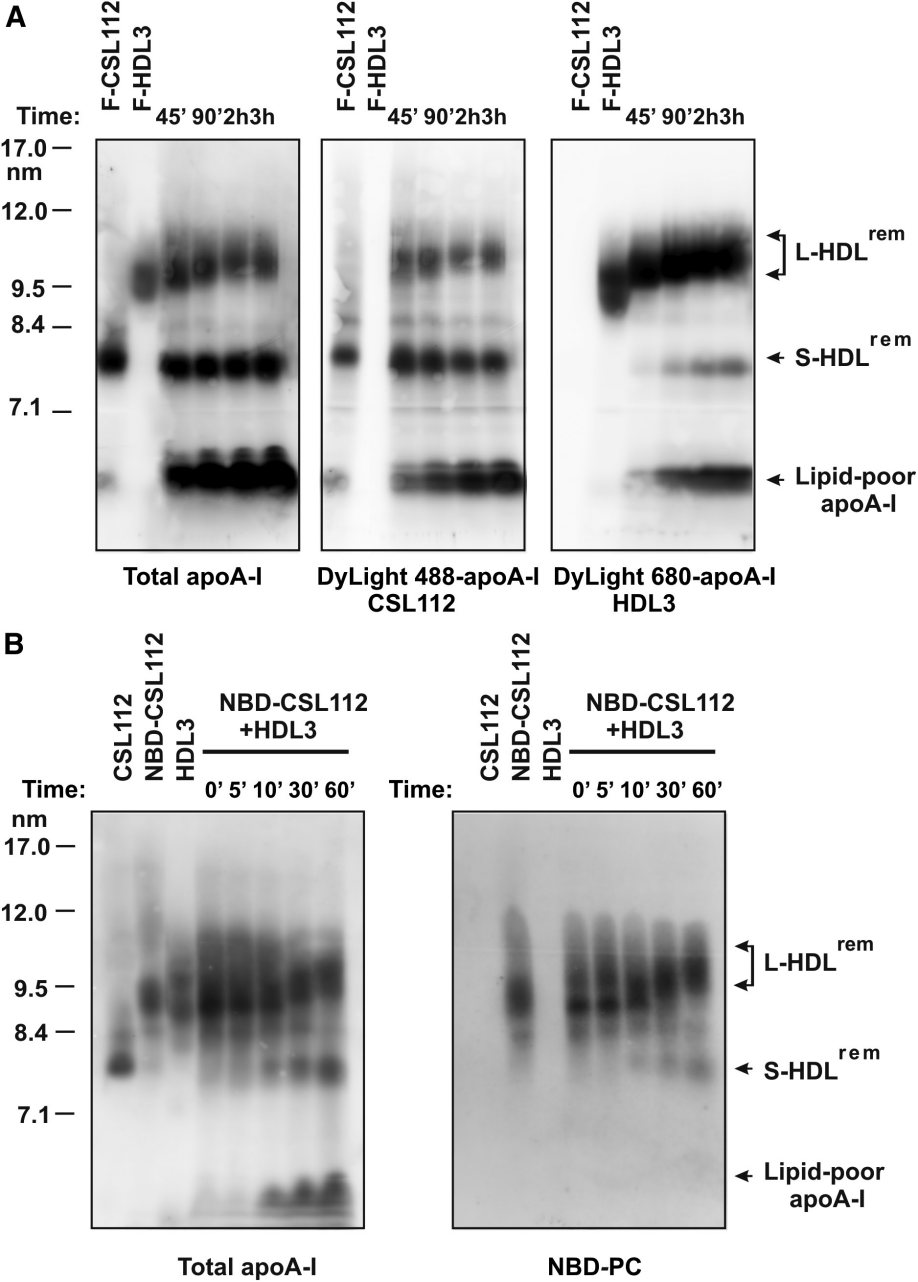
**Dynamics of transfer of apolipoprotein A-I (apoA-I) and lipid content during particle remodeling. A**, Fluorescently labeled CSL112 (DyLight488-CSL112) and high-density lipoprotein (HDL)3 (DyLight680-HDL3) were incubated at 37°C for the indicated periods of time. DyLight488-apoA-I (**middle**) and DyLight680-apoA-I (**right**) were visualized by fluorescence imaging of the blots, total apoA-I content (left panel) was analyzed by Western blotting with an anti–apoA-I antibody. **B**, CSL112-derived phospholipid is transferred to HDL on remodeling. CSL112 was labeled with a fluorescent phospholipid analog (18:1–06:0 NBD-PC). NBD-PC–labeled CSL112 (NBD-CSL112) was incubated with purified HDL3 for the indicated periods of time at 37°C. Lipoprotein mixtures were subjected to nondenaturing polyacrylamide gradient gel electrophoresis and analyzed by Western blotting with an anti–apoA-I antibody (**left**). NBD-PC was visualized by fluorescence imaging of the blots (**right**). The positions of migration of remodeled HDL subspecies, L-HDL^rem^ and S-HDL^rem^, and lipid-poor apoA-I are indicated.

To closely follow the dynamics of the particle remodeling, we used CSL112 labeled with DyLight 488 fluorophor and HDL3 labeled with DyLight 680 fluorophor for the incubation, and monitored the distribution of specifically labeled apoA-I among the products of the remodeling over time, from 45 minutes to 3 hours. Fluorescence imaging revealed that both DyLight 488 apoA-I and DyLight 680 apoA-I derived from CSL112 and HDL3, respectively, progressively accumulated in lipid-poor apoA-I in a continuous, time-dependent manner (Figure 4A). Formation of large HDL subspecies (L-HDL^rem^) was characterized by a rapid association of parent HDL3 with DyLight 488 apoA-I from CSL112, but its abundance in large HDL subspecies remained unchanged. S-HDL^rem^ particles were primarily composed of DyLight 488 apoA-I after 45 minutes of incubation confirming that they originate from CSL112. During prolonged incubation (between 90 minutes and 3 hours), DyLight 488 apoA-I was partially displaced by DyLight 680 apoA-I from HDL3 in a time-dependent manner, suggesting ongoing interaction between S-HDL^rem^ and L-HDL^rem^. Such ongoing interaction is also suggested by the continued growth in the size of L-HDL^rem^ during this period (Figure 4A, right).

Next, we examined whether phospholipid transfer between CSL112 and HDL may contribute to the decline of CSL112 size during conversion to S-HDL^rem^ and increase in size of HDL3 during conversion to L-HDL^rem^. CSL112 was labeled with a fluorescent phospholipid analog (18:1–06:0 NBD-PC). After labeling, the molar ratio of phosphatidylcholine/apoA-I in NBD-PC–labeled CSL112 was increased compared with that in unlabeled CSL112 (72:1 and 50:1, respectively). Incubation of HDL3 with NBD-PC–labeled CSL112 resulted in a rapid movement of the major portion of CSL112-derived fluorescent lipid into L-HDL^rem^ (Figure 4B, right). S-HDL^rem^ showed low levels of the fluorescent lipid deriving from CSL112. Thus, remodeling of CSL112 to S-HDL^rem^ involves retention of apoA-I (Figure 4A, left) but loss of phospholipid (Figure 4B, right) and transfer of the phospholipid to L-HDL^rem^.

These data further support remodeling by a transient fusion of CSL112 and HDL (Online Figure VII). Subsequent fission yields lipid-poor apoA-I composed of apoA-I from both CSL112 and HDL3, and enlarged HDL3 (L-HDL^rem^) particles made larger by receipt of both apoA-I and phospholipid from CSL112. The third product, S-HDL^rem^, seems to be CSL112, which has lost some of its phospholipid to HDL3. This model was further tested by compositional analysis of the isolated products of remodeling.

### Lipid Composition, Morphology, and Structure of the Individual Products of HDL Remodeling

The 3 individual products of remodeling, L-HDL^rem^ (≈10–11 nm), S-HDL^rem^ (≈ 7.3–7.6 nm), and lipid-poor apoA-I (≈5.4 nm), were isolated out of the incubation mixture of CSL112 and HDL3 by preparative ultracentrifugation. Particle purity was analyzed by gradient gel electrophoresis (Online Figure VIII), particle content of choline-containing phospholipids was measured enzymatically and by high-performance liquid chromatography, and data were expressed as the lipid/total protein ratio (Online Table I). We observed that the phospholipid/total protein ratio of L-HDL^rem^ was significantly increased (2-fold) compared with parent HDL3, whereas for the S-HDL^rem^, this ratio was decreased (2.3-fold) compared with parent CSL112. The amount of phospholipid measured in lipid-poor apoA-I was low and close to the detection limit (Online Table I).

Next, phospholipid content of CSL112, HDL3 and purified products of the remodeling was analyzed by mass spectroscopy. The fatty acid chain lengths of the phosphatidylcholine in CSL112 differ from those in HDL3 (Online Table II), in particular, phosphatidylcholine (18:2/18:2) is abundant in CSL112 but rare in HDL3. This fatty acid signature of phospholipid originally present in CSL112 was found at high levels in L-HDL^rem^ (Table), confirming studies with NBD-phosphatidylcholine (Figure 3B) showing that HDL particle remodeling is accompanied by phospholipid transfer from CSL112 to HDL3. In a similar fashion, phospholipid species, such as phosphatidylinositol, that are present in HDL but not in CSL112 allowed us to document movement of phospholipids from HDL3 into S-HDL^rem^ (Table; Online Table II). Despite the movement of HDL3-derived phospholipid to S-HDL^rem^ during remodeling, the net movement of phospholipid is from CSL112 into HDL3 and contributes to the increase in size of the large remodeled product (L-HDL^rem^). The net loss of phospholipid by CSL112 seems to account for its reduction in size and transformation into S-HDL^rem^.

**Table.**
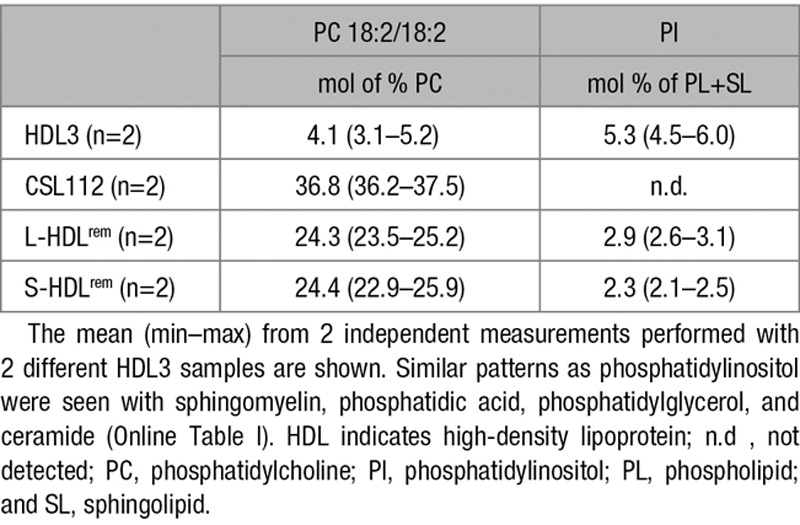
Phospho- and Sphingolipid Composition of Native, Reconstituted, and Remodeled HDL Species

To examine morphology and structure of the products of HDL particle remodeling, we used an optimized electron microscopy method that minimizes rouleau formation and enables better visualization and measurements of individual lipoprotein particles. We measured the geometric mean of longest diameter (representing particle size), its orthogonal diameter, and their aspect ratio (representing particle shape).

**Figure 5.**
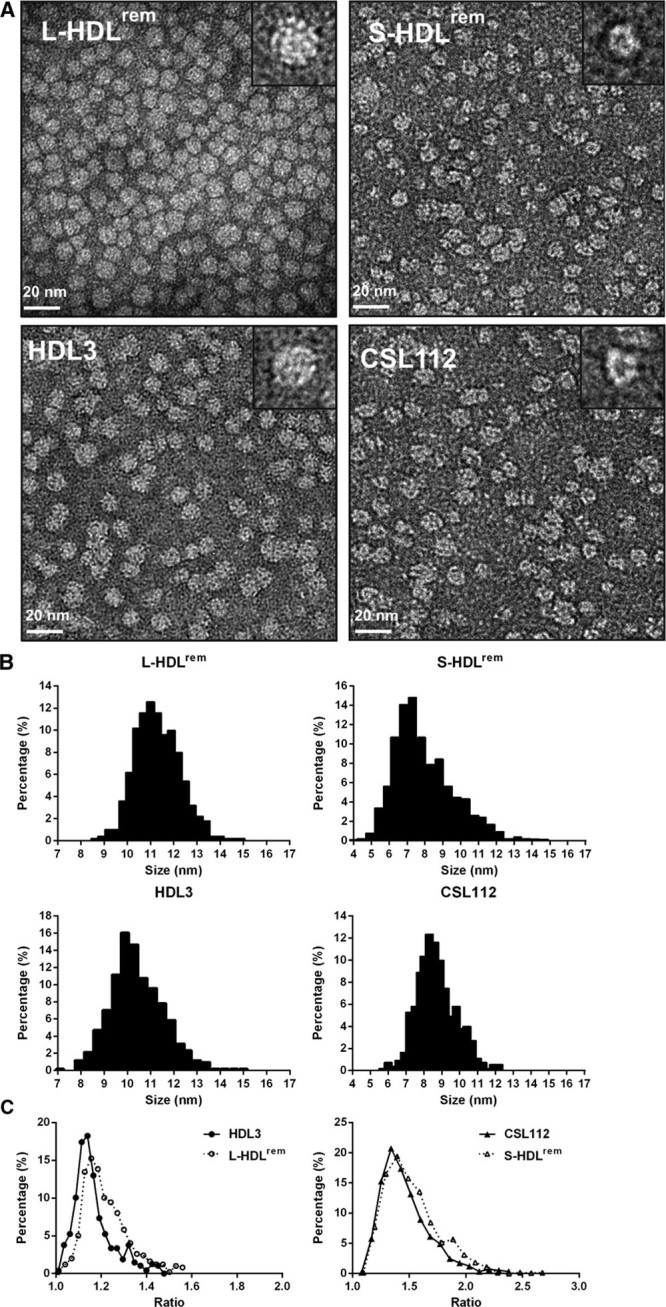
**Electron microscopy analysis of the structure and morphology of isolated individual products of high-density lipoprotein (HDL) remodeling.** Samples of remodeled HDLs (large [L-HDL^rem^] and small [S-HDL^rem^] species purified from CSL112 and HDL3 incubation mixtures), HDL3 and CSL112 were prepared with the optimized NS-EM protocol using uranyl acetate as the negative stain. **A**, Micrographs for each sample tested are shown with selected individual particles shown on the inserts (particle window size=20 nm). Particle size and shape distribution were analyzed (**B** and **C**). The number of particles analyzed was n=501 for L-HDL^rem^, n=533 for S-HDL^rem^, n=511 for HDL3, and n=551 for CSL112. Particle size distribution (**B**) was measured as the geometric mean of 2 perpendicular diameters (longest and shortest). Particle shape distribution (**C**) was measured as the aspect ratio between them.

On electron micrographs, HDL3 and L-HDL^rem^ seem as spherical particles with contiguous high densities near the particle’s edge and center. In contrast, CSL112 and S-HDL^rem^ seem as flattened ovoid discs without rouleau (Figure 5A). Computed geometric means for HDL3 and L-HDL^rem^ showed that the L-HDL^rem^ particles were larger compared with HDL3. The majority of L-HDL^rem^ particles were between 9 and 14 nm in diameter, with the peak population (≈12%) at ≈11.0 nm, whereas HDL3 particles were 7 to 13 nm in diameter, with the peak population (≈16%) at ≈9.8 nm (Figure 5B). The peak population of L-HDL^rem^ and HDL3 had aspect ratios of 1.15 and 1.14, respectively, confirming their spherical morphology (Figure 5C).

In contrast with HDL3 and L-HDL^rem^, CSL112 and S-HDL^rem^ particles appear as contiguous irregular cable-like, high-density rings, with their central regions appearing as a lower density hollow (Figure 5A). The geometric means for CSL112 particles were between 7 and 11 nm and the peak population (≈12.4%) at 8.4 nm. S-HDL^rem^ particles were smaller, with the majority of particles being between 5 and 10 nm in diameter and the peak population (≈14.9%) at 7.3 nm (Figure 5B). Comparison of aspect ratios showed similar features for S-HDL^rem^ and CSL112 particles. The peak population of S-HDL^rem^ and CSL112 had aspect ratios of 1.39 and 1.33, respectively (Figure 5C), suggesting a discoidal morphology. Electron microscopy analysis of lipid-poor apoA-I did not reveal any particular structures, globular or discoidal (data not shown).

### Enhanced Cholesterol Efflux and Anti-Inflammatory Effects Mediated by CSL112 Are Linked to HDL Particle Remodeling

**Figure 6.**
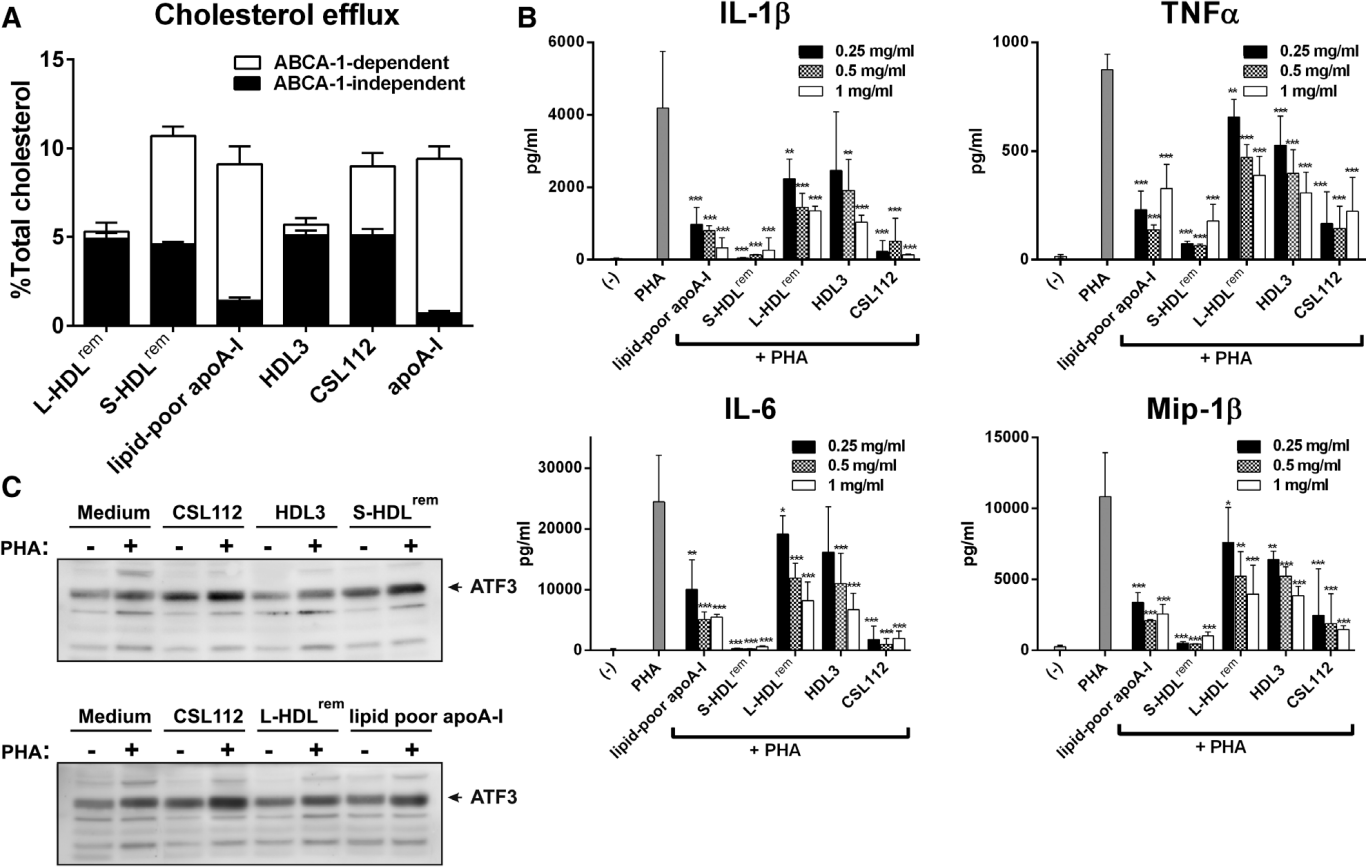
**Enhanced cholesterol efflux and anti-inflammatory effects mediated by CSL112 are linked to high-density lipoprotein (HDL) particle remodeling. A**, ATP-binding cassette transporter 1 (ABCA1)–dependent and ABCA1-independent cholesterol efflux from RAW264.7 cells to isolated individual products of HDL remodeling (large [L-HDL^rem^] and small HDL species [S-HDL^rem^], and lipid-poor apolipoprotein A-I [apoA-I]). Final concentration of cholesterol acceptors in efflux medium was 20-μg protein/mL. Each fractional efflux value represents the mean±SD for 3 independent experiments performed with different samples measured in triplicate. **B**, Small HDL species inhibit proinflammatory cytokine production in peripheral blood mononuclear cells (PBMCs). Tumor necrosis factor-α (TNF-α), interleukin-1β (IL-1β), IL-6, and macrophage inflammatory protein-1β (Mip-1β) were measured in cell-free supernatants at 20 h after phytohemagglutinin (PHA) stimulation. The mean values±SD are shown and are derived from triplicate cell cultures of at least 4 donors. ***P<0.001, **P<0.01, *P<0.05 vs control PHA-stimulated cells. **C**, Small HDL species stimulate activating transcription factor 3 (ATF3) in PHA-M–stimulated PBMC. PBMC were stimulated or not with PHA-M (1 µg/mL) in the presence of different lipoproteins (at final protein concentration of 1 mg/mL). Cellular lysates were subjected to SDS-PAGE followed by Western blotting with an anti-ATF3 antibody.

We have previously shown that CSL112 improves HDL functionality in plasma, increasing the capacity to support cholesterol efflux and to inhibit proinflammatory cytokine production.^19^ To evaluate the contribution of HDL remodeling induced by CSL112 to the increase in HDL functionality, we examined the functional properties of the individual remodeled HDL subspecies using CSL112 and HDL3 for direct comparison. Two products of HDL remodeling, lipid-poor apoA-I and the S-HDL^rem^, exhibited a much higher capacity to efflux cholesterol via ABCA1 compared with the original CSL112 and HDL3 or L-HDL^rem^. In sharp contrast, almost no ABCA1-independent efflux was promoted by lipid-poor apoA-I. S-HDL^rem^ enhanced ABCA1-independent efflux to a similar degree compared with other lipidated HDL particles tested (CSL112, HDL3, or L-HDL^rem^; Figure 6A). Additional studies using a different cell system confirmed these findings about ABCA1 (Online Figure IX) and further showed that both S-HDL^rem^ and L-HDL^rem^ are good acceptors of cholesterol via ATP-binding cassette subfamily G member 1 while, as expected by their low lipid content, S-HDL^rem^ and lipid-poor apoA-I were poor acceptors of cholesterol via scavenger receptor class B member 1. Taken together, these data indicate that the process of remodeling leads to the formation of 2 HDL subpopulation, lipid-poor apoA-I and S-HDL^rem^, which are extremely efficient acceptors of cholesterol via ABCA1 pathway while efflux via ATP-binding cassette subfamily G member 1 and scavenger receptor class B member 1 is maintained. Importantly, this process seems driven by the admixture of lipoprotein particles because incubation of CSL112, HDL2, or HDL3 alone did not lead to increases in efflux activity (Online Figure VI). Thus, an increase in concentration of these HDL subpopulation driven by CSL112 infusion in vivo may account for the elevated capacity of plasma to induce cholesterol efflux via ABCA1.

Next, we examined the ability of remodeled HDL subspecies to inhibit phytohemagglutinin-induced proinflammatory cytokine production in human PBMC. Lipid-poor apoA-I, S-HDL^rem^, and CSL112 exerted the strongest inhibitory effects on secretion of proinflammatory mediators (tumor necrosis factor-α, IL-1β, IL-6, and macrophage inflammatory protein-1β), whereas HDL3 and L-HDL^rem^ were much less effective (Figure 6B). The extent of inhibition by the products of HDL remodeling in PBMC correlated positively with the induction of activating transcription factor 3 (ATF3), a known negative regulator of the macrophage transcriptional response to inflammatory stimuli.^26,27^ Lipid-poor apoA-I and S-HDL^rem^ induced higher protein levels of ATF3 in phytohemagglutinin-stimulated PBMC compared with medium control, HDL3 or L-HDL^rem^ (Figure 6C).

### Antioxidative Activity of the Products of HDL Remodeling

**Figure 7.**
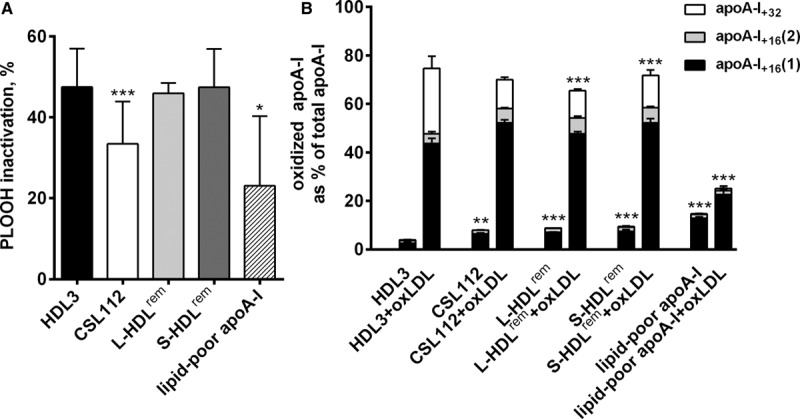
**On remodeling, antioxidative activity was predominantly associated with large and small remodeled high-density lipoprotein (HDL) species, L-HDL^rem^ and S-HDL^rem^, paralleled by the increase in oxidation of** apolipoprotein A-I (**apoA-I) Met residues. A**, Inactivation of phospholipid hydroperoxide (PLOOH) in oxidized LDL (oxLDL) incubated with HDL3, CSL112, or individual products of HDL remodeling. LDL (40 mg total cholesterol [TC/dL]) was oxidized by 2,2′-Azobis(-amidinopropane) dihydrochloride (4 mol/L) for 6 h, dialyzed and incubated at 20 mg/TC/dL alone or in the presence of individual lipoproteins (each at a final concentration of 8 mg total protein/dL) for 4 h at 37°C. PLOOH was evaluated in incubation mixtures by high-performance liquid chromatography (HPLC) with chemiluminescent and ultraviolet (UV) detection. Inactivation of PLOOH in oxLDL+HDL incubation mixtures is presented as percentage change from the PLOOH levels detected in oxLDL incubated alone. Means±SDs for at least 3 independent experiments are shown; ***P<0.001, *P<0.05 vs nonmodified HDL3 (HDL3). **B**, Oxidation of apoA-I Met residues in different HDL species incubated alone or with oxLDL. Oxidized at Met residues and nonoxidized apoA-I were measured by HPLC using UV detection. ApoA-I_+16_ and apoA-I_+32_ refer to oxidized forms 16 and 32 U greater, respectively, than nonoxidized apoA-I as shown by mass spectrometry. ApoA-I_+32_, apoA-I oxidized at Met112 and Met86; ApoA-I_+16_, apoA-I oxidized at Met112 (1) or Met86 (2).

HDL has been shown to protect LDL from oxidative damage by inhibiting accumulation of primary and secondary peroxidation products in LDL.^28^ To evaluate antioxidative activity of the products of HDL remodeling, we compared their capacity to inactivate PLOOH in LDL preoxidized by 2,2′-Azobis(2-amidinopropane) dihydrochloride (oxLDL). HDL3, L-HDL^rem^, and S-HDL^rem^ equally well attenuated PLOOH content in incubation mixtures with oxLDL (47.5±9.5%, 45.9±2.6%, and 47.4±9.5% PLOOH inactivated, respectively), whereas CSL112 and lipid-poor apoA-I were markedly less effective (33.4±10.5% and 23.1±2.6% PLOOH inactivated, respectively, Figure 7A). HDL-mediated inactivation of linoleic acid 9-mono hydroperoxide (LOOH) was associated with the formation of oxidized forms of apoA-I characterized by the conversion of apoA-I Met 112 and Met 86 residues into Met(O) (Figure 7B). Oxidation of apoA-I in different HDL species after incubations with oxLDL decreased in the order HDL3=S-HDL^rem^≥L-HDL^rem^>CSL112>lipid-poor apoA-I, thereby mirroring their protective activity during LDL oxidation (Figure 7A).

As absolute concentrations of apoA-I in HDL samples tested were similar, low antioxidative activity of lipid-poor apoA-I suggests that because of structural characteristics of this particle, oxidation-sensitive Met residues were poorly accessible for the interaction with PLOOH. Overall, a positive correlation observed between the levels of oxidized apoA-I and levels of inactivated PLOOH supported a role for Met residues of apoA-I as the principal mechanism of PLOOH inactivation by HDL.

## Discussion

In earlier work, we showed that addition of CSL112 to plasma dramatically elevates cholesterol efflux capacity of human serum.^19^ Careful quantitation showed that CSL112 elevated efflux capacity of plasma much more strongly than an equivalent amount of HDL3. Similarly, the anti-inflammatory effects of addition of CSL112 to whole human blood were substantially stronger than those caused by addition of HDL3.^19^ Here, we show that this enhancement of HDL function by CSL112 may be ascribed to particle remodeling with formation of small, highly functional species. The small forms, lipid-poor apoA-I and S-HDL^rem^, produced during remodeling, exhibit both an ABCA1-dependent efflux capacity and an anti-inflammatory capacity that are higher than that of HDL3 or large remodeled HDL (L-HDL^rem^). This increased activity may thereby explain the rise in functional HDL, that is greater than the rise in HDL-C or apoA-I. Such remodeling occurs on infusion of CSL112 into human subjects, addition of CSL112 to plasma, and on incubation of CSL112 with purified HDL. The correlation of CSL112-driven remodeling with cholesterol efflux capacity observed here is consistent with the findings of Borja et al^29^ who found a strong correlation between the exchangeability of apoA-I on plasma HDL and the cholesterol efflux capacity of plasma.

We have purified and analyzed the function of 2 small species: the lipid-poor apoA-I and S-HDL^rem^ and found that both species are individually active in ABCA1-mediated cholesterol efflux and in reducing the cytokine response of blood leukocytes to an inflammatory stimulus (Figure 6A and 6B). To our knowledge, this is the first functional analysis of these species in isolation. The potent capacity of small, dense HDL subspecies to efflux cellular cholesterol through ABCA1 is fully consistent with recent demonstration of HDL particle size being a critical determinant of ABCA1-mediated cholesterol export from macrophages, with a HDL particle size equal to 8.0 nm or lower being required for efficient cholesterol efflux through ABCA1.^6^ The correlation of anti-inflammatory activity with ABCA1-dependent efflux activity is consistent with the view that cholesterol depletion may cause the anti-inflammatory effects.^30^ These findings are in keeping with published reports demonstrating that cholesterol efflux is required to prevent cell activation.^31,32^

We have also confirmed the findings of De Nardo et al^27^ that reconstituted HDL causes induction of ATF3, a negative transcription regulator of toll-like receptor-induced cytokine production in macrophages^26^ and have expanded the findings by showing that while large HDL species have low ability to induce ATF3, it is the smaller species that have the strongest anti-inflammatory and ATF3-inducing capacity (Figure 6B and 6C). ATF3 has been shown to regulate lipid body formation in macrophages, at both basal and high levels of cellular lipid loading, representing an intersection point for inflammatory and metabolic pathways in macrophages.^33^ Thus, infusion of CSL112 and the resulting increase of small HDL species, S-HDL^rem^ and lipid-poor apoA-I, capable of inducing ATF3 may be beneficial to modulate both foam cell formation and inflammation via ATF3.

Studies of the antioxidant activity of the remodeled HDL species showed that while S-HDL^rem^ was capable of reducing the PLOOH content of oxLDL, the lipid-poor apoA-I displayed low activity. This finding is consistent with previous data documenting potent capacity of small, dense HDL subspecies from human plasma to protect LDL from oxidative stress,^28^ and to inactivate PLOOH derived from oxLDL.^23^ Mechanistically, the antioxidative effects of HDL on LDL lipids involve transfer to HDL of their oxidized species, primarily those derived from phospholipids, with subsequent inactivation.^34^ The transfer process depends on the fluidity of surface HDL lipids and may require the presence of an organized lipid surface in HDL; as the latter is most likely not the case in lipid-free apoA-I, this structural feature can account for its low capacity to inactivate PLOOH. Together, the present data on the potent biological activities of remodeled small, dense HDL particles provide further support to the notion of distinct role of particle subspecies in the protection from atherosclerosis.^28,35^

Contrary to our expectations, HDL remodeling induced by CSL112 was independent of plasma lipid transfer proteins or lipases, as heat inactivation of plasma, inhibition of CETP, LCAT, secretory phospholipase 2 with specific inhibitors or stimulation of PLTP, or genetic deletion of PLTP or LCAT neither prevented nor accelerated the formation of lipid-poor apoA-I (Online Figures IV and V). Rather, remodeling appeared to arise from a spontaneous interaction of CSL112 with HDL2 and HDL3. We speculate that this interaction involves first a fusion of CSL112 with native HDL and a subsequent fission to yield 3 remodeling products. An initial intimate interaction between CSL112 and HDL is indicated first by the observation of bulk movement of phospholipid from CSL112 to the larger remodeled product and by the smaller inverse movement of HDL3-derived phospholipid to the smaller remodeled product (Table; Online Tables I and II). Phospholipid has little ability to diffuse in aqueous solutions, thus favoring the notion of initial particle fusion. The suggestion of fusion between CSL112 and native HDL and subsequent fission is further supported by analysis of the size, and protein origin, and lipid constituents of the reaction products (Figures 3 and 4).

Fusion of particles with subsequent release of lipid-poor apoA-I has also been postulated to be the mechanism by which PLTP promotes the formation of lipid-poor apoA-I.^18^ Although these authors proposed a direct bridging role for PLTP in the fusion, the data reported here suggest an alternative path: PLTP-mediated exchange of lipids among lipoprotein particles may give rise to HDL species with structure similar to CSL112, which spontaneously fuse with HDL2 or HDL3. Indeed, Ji et al^36^ have shown that pretreatment of apoA-I with PLTP enables it to bind rapidly to HDL2 or HDL3.

The fission of a fused CSL112-HDL particle may be a physical consequence of its composition. Because the disc-shaped CSL112 contains only surface lipids and no core lipids (neither triglycerides nor cholesterylester), then the products of fusion will of necessity have an increased ratio of surface to core. This relative excess of surface may promote dissociation of apoA-I by a process analogous to that which may occur during lipolysis of a chylomicron. Nevertheless, the initial interaction of CSL112 and HDL3 seems to have more than 1 potential outcome (Online Figure VII). During the course of remodeling, we observed formation not only of lipid-poor apoA-I but also of a species smaller than CSL112 and with less phospholipid (S-HDL^rem^). We speculate that S-HDL^rem^ is mainly a product of a net transfer of phospholipids from CSL112 to HDL3. This is consistent with the finding that both CSL112 and S-HDL^rem^ particles seem to be of a discoidal morphology, but particle size of S-HDL^rem^ is smaller than that of CSL112 (Figure 5). Supporting this deduction is also the observation that while CSL112 seems to rapidly donate phospholipid to HDL3, the movement of apoA-I from HDL3 into S-HDL^rem^ occurs much more slowly (Figure 4). Similar observations have been made by Settasation et al^18^ who showed that a fusion product of spherical HDL particles may release daughter particles of either small or intermediate size. Finally, we note that S-HDL^rem^ particles seem to retain the ability to fuse with endogenous HDL as they gradually exchange apoA-I with L-HDL^rem^ and eventually disappear during longer incubation in plasma (Figure 2A).

The main limitation of the current work is that the detailed studies of remodeling were done in vitro under cell-free conditions and, thus, the contribution of cell-bound enzymes, such as lipases, cannot be taken into account. In addition, it is expected that in vivo, cholesterol from blood cells and other tissues may join the lipoprotein pool and affect this remodeling. Nevertheless, we saw no change in the kinetics of remodeling in plasma versus whole blood (Online Figure X) versus in man (Online Figure III). CSL112 did promote remodeling of HDL2 and HDL3 with slightly different kinetics of lipid-poor apoA-I formation (Figure 3A), suggesting that differences in particle size may have potential to influence HDL particle remodeling. Protein composition is also likely to affect fusion, and previous work has shown that apoA-II inhibits CETP-mediated remodeling of reconstituted HDL into large and small particles in a process that leads to lipid-poor apoA-I formation.^37^ Analysis of apoA-II distribution among the products of CSL112-induced remodeling of HDL3 revealed that apoA-II is present in remodeled large HDL particles with its time-dependent distribution pattern identical to that of apoA-I, but it is absent in remodeled small HDL and lipid-poor apoA-I (Online Figure XI), and infusion of CSL112 did not significantly change apoA-II blood levels (Online Figure II). These data suggest that while apoA-II did not inhibit fusion with CSL112, it did not join apoA-I in forming smaller fission products. Finally, ApoE seems unlikely to play a role in the remodeling described here because neither CSL112 nor HDL3 bear apoE (Online Figure XII), yet CSL112 drives remodeling of HDL3. Furthermore, CSL112 did not affect the size distribution of apoE-containing particles in plasma (Online Figure I).

We have used an exogenously supplied source of apoA-I, CSL112, to document a novel mechanism for formation of small forms of HDL with high biological activity. We speculate that this process may occur not only after infusion of a reconstituted HDL but also during the normal lifecycle of HDL. After synthesis, lipid-poor apoA-I is lipidated by ABCA1 and gives rise to discoidal nascent HDL.^38^ Our work suggests that one fate of such particles may involve fusion with spherical HDL and production of smaller daughter products. Such a process of fusion of nascent discs with circulating spheres may underlie the recent findings of Mendivil et al^39^ who observed direct hepatic secretion of all size classes of HDL in the venous blood of human volunteers. Our data suggest that fusion of nascent discs made by the liver with native HDL in the space of Disse or hepatic sinusoids would lead to rapid appearance of newly synthesized apoA-I in all size classes, as observed by Mendivil et al.^39^

It has been long recognized that HDL may exist in forms with higher or lower functional activity. Recent observations now strongly connect HDL function with coronary heart disease outcomes^2–4^ and make understanding the factors that change HDL functionality a vital topic. Here we characterize highly active HDL species and describe a novel mechanism for their formation in response to CSL112, a product currently in clinical development for prevention of early recurrent atherothrombotic events in acute coronary syndrome. Importantly, ongoing clinical evaluation of CSL112 holds the hope of validating the connection between functional assays such as those studied here and clinical disease.

## Acknowledgments

We would like to thank Drs Rye and Remaley for careful reading of the article and critical comments. We gratefully acknowledge Dr Jiang for providing plasma samples of wild-type and PLTP knockout mice, B. Haenni, M. Kaminek, and Dr B. Zuber for electron microscopy analysis performed at the Microscopy Imaging Center (MIC) of the University of Bern and Dr Asztalos for 2-dimensional gel analysis. Editorial assistance with the preparation of this article was provided by Meridian HealthComms Ltd, funded by CSL Behring.

## Sources of Funding

This work was funded by CSL Behring.

## Disclosures

S.A. Didichenko, A.V. Navdaev, A. Gille, P. Schuetz, M.O. Spycher, and S.D. Wright are employees of CSL Behring or CSL Limited. M.J. Chapman is a member of Advisory Board and Speakers Bureau: Amgen, Sanofi-Regeneron, Unilever, Astrazeneca. M.J. Chapman and A. Kontush are coauthors of a patent on the enhancement of HDL function by phosphatidylserine. The other authors report no conflicts.

## Supplementary Material

## Novelty and Significance

### What Is Known?

- Patient-to-patient variation in cholesterol efflux capacity, a known predictor of coronary heart disease risk, is driven primarily by differences in ATP-binding cassette transporter–-dependent efflux, a process dependent on small but not large high-density lipoprotein (HDL).
- CSL112 is human apolipoprotein A-I reconstituted with phosphatidylcholine, currently in development to treat acute myocardial infarction.
- Infusion of CSL112 elevates ATP-binding cassette transporter–-dependent efflux and causes remodeling of HDL with a very dramatic rise in small HDL.
