# Supplementary material for: Enhanced HDL Functionality in Small HDL Species Produced Upon Remodeling of HDL by Reconstituted HDL, CSL112: Effects on Cholesterol Efflux, Anti-Inflammatory and Antioxidative Activity
Source: Circ Res. 2016 Sep 1;119(6):751–63. doi: 10.1161/CIRCRESAHA.116.308685 (PMC5006797; doi:10.1161/CIRCRESAHA.116.308685)
Supplement: Supplementary file 1 [file res-119-751-s001.pdf]

Circulation Research

From: Asztalos, Bela <Bela.Asztalos@tufts.edu>
Sent: Wednesday, July 06, 2016 8:52 AM
To: circulation.research@circresearch.com
Cc: Kate.Holliday@meridian-health.com
Subject: permission

I give permission to the Circulation Journal for using my name in the article titled "**Enhanced HDL functionality in small HDL species produced upon remodeling of HDL by reconstituted HDL, CSL112: effects on cholesterol efflux, anti-inflammatory and antioxidative activity"**
Sincerely,
Bela Asztalos
