# Supplementary material for: Enhanced HDL Functionality in Small HDL Species Produced Upon Remodeling of HDL by Reconstituted HDL, CSL112: Effects on Cholesterol Efflux, Anti-Inflammatory and Antioxidative Activity
Source: Circ Res. 2016 Sep 1;119(6):751–63. doi: 10.1161/CIRCRESAHA.116.308685 (PMC5006797; doi:10.1161/CIRCRESAHA.116.308685)
Supplement: Supplementary file 2 [file res-119-751-s002.pdf]

SUPPLEMENTAL MATERIAL

Methods

HDL labeling

Biotinylation of CSL112 or high-density lipoprotein 3 (HDL3) was performed as described by Lund-Katz *et al*[1] with modifications. Briefly CSL112 or HDL3 were dialyzed into PBS (pH 7.4) prior to biotinylation. The EZ-link sulfo-NHS-LC-biotinylation kit (Pierce Biotechnology) was used for attaching biotin molecules through a 2.24 nm spacer arm to lysine residues on the surface of HDL particles. CSL112 or HDL3, each at 10 mg protein/mL, were mixed with 10 mmol/L sulfo-NHS-LC-biotin solution at a 30-fold molar excess of biotin. The lipoproteins were incubated at 4°C for 30 min, reaction was terminated by adding glycine (pH 7.5) up to final concentration of 100 mM for 30 min. Unbound biotin was removed by ultrafiltration using Amicon Ultra Centrifugal Filter (Ultracel 50K; Merck Millipore). The degree of biotinylation of the particles was determined using conditions recommended by Pierce Biotechnology. For fluorescence imaging, CSL112 or HDL3 were labeled with DyLight 488 or DyLight 680 Amine-Reactive Dyes (Pierce Biotechnology) following the manufacturer's recommendations. CSL112 or HDL3, each at 5 mg protein/mL, were mixed with DyLight fluorophore solution at a 5.5-fold molar excess of fluorophore. The labeling procedures performed yielded one to two biotin or DyLight molecules per apoA-I molecule in CSL112 or HDL particles.

CSL112 was labeled with a fluorescent phospholipid analog (18:1-06:0 NBD PC [1-oleoyl-2-{6-[(7-nitro-2-1,3-benzoxadiazol-4-yl)amino]hexanoyl}-*sn*-glycero-3-phosphocholine], Avanti Polar Lipids) as previously described.[2] CSL112 was diluted in PBS (final concentration of 2 mg protein/mL) and incubated with the NBD-PC-precoated glass beads with agitation at room temperature for 3 h. After pelleting the beads, NBD-PC-labeled CSL112 was subjected to ultracentrifugation at a density of 1.125 g/mL for 20 h at 10°C (55,000 rpm, TFT 70.38 rotor). Unbound NBD-PC micelles concentrated in a layer at the top of the tube were removed. The density of the bottom fraction containing NBD-PC-labeled CSL112 was readjusted and centrifugation was repeated to ensure complete removal of unbound NBD-PC micelles. NBD-PC-labeled CSL112 was concentrated with Amicon Ultra Centrifugal Filter (Ultracel 100K; Merck Millipore) and dialyzed against PBS. Due to labeling, the ratio of PC/apoA-I (mol/mol) in NBD-PC-labeled CSL112 increased compared to the unlabeled CSL112, 72:1 and 50:1, respectively.

In vitro incubations

For the incubation of CSL112 with human whole blood or plasma, blood was collected from healthy donors into EDTA vacutainer tubes. Plasma was prepared by centrifugation of blood at 700 *g* for 20 min at 4°C. Before incubation with CSL112, plasma was diluted with 0.9% NaCL to adjust for the volume of pelleted red blood cells. CSL112 (stock concentration of 10 mg protein/mL) was added to 190 μL of whole blood or plasma to a final concentration of 1 mg protein/mL and incubation was carried out for the different periods of time (10 min, 30 min, 1 h and 4 h) at 37°C. At the end of incubation, blood samples were briefly centrifuged to obtain cell-free supernatants. All samples were snap frozen in liquid nitrogen and stored at -70°C until required for analysis. To remove apo-B-containing lipoproteins, plasma or blood–derived samples were precipitated with polyethylene glycol (PEG). Briefly, samples were mixed with 0.4 volumes of 20% PEG 8000 MW (Sigma P-2139) in 200 mM glycine pH 7.4 and centrifuged at 14 000 rpm for 5 min at 4°C. Supernatants containing HDL fraction were collected and used for electrophoretic analysis and for the measurements of cholesterol efflux from RAW264.7 cells.

Plasma samples of wild-type (WT), LCAT KO[3] and PLTP KO[4] mice in a C57BL/6 background were used for the incubation with CSL112. CSL112 was incubated with mouse plasma at a final concentration of 0.75 mg protein/mL for 1h at 37°C. For electrophoretic analysis, amount of plasma protein loaded on a gel was equivalent to 2 μL of undiluted plasma (wild-type mice), and 4 μL of undiluted plasma (PLTP KO and LCAT KO mice).

Lipoprotein isolation

LDL, HDL2 and HDL3 were isolated from cryo-depleted plasma (CSL Behring) by sequential ultracentrifugation.[5] Briefly, plasma was adjusted to appropriate densities by adding solid KBr. Lipoproteins were isolated using a TFT 70.38 rotor at a speed of 55,000 rpm. LDL (d=1.019-1.055 g/mL), HDL2 (*d* =1.065-1.121 g/mL) and HDL3 (*d* =1.13-1.18 g/mL) were isolated with a single spin of 24 h at the lower density followed by successive spin for the same period at the higher density. The isolated lipoproteins were dialyzed against endotoxin-free phosphate buffered saline (PBS) [pH 7.4].

Incubation of CSL112 with individual lipoproteins was carried out in PBS or in PBS supplemented with 0.5% fatty acid free bovine serum albumin (BSA, Sigma Aldrich), at a concentration of 1 mg protein/mL of each lipoprotein.

For preparative isolation of the products of HDL remodeling, CSL112 was incubated with HDL3 in PBS for 1 hour at 37°C. Two lipoprotein fractions were purified from the incubation mixtures by sequential ultracentrifugation in the density intervals: remodeled large HDL subspecies (Fr. 1; *d* =1.125-1.224 g/mL) and remodeled small HDL subspecies (Fr. 2; *d* =1.22-1.24 g/mL). In each case samples were subjected to two successive spins for 20-22 h at 10°C (55,000 rpm, TFT 70.38 rotor), first spin at the higher density followed by the spin at the lower density. Lipid-poor apoA-I (Fr. 3) was collected at the bottom of the tube following centrifugation at the density *d* =1.24 g/mL. The final preparations were dialyzed against endotoxin-free PBS (pH 7.4) using Mini Dialysis Kit (molecular weight cut off 8 kDa; GE Healthcare). Amicon Ultra Centrifugal Filters (Ultracel 30K and 100K; Merck Millipore) were used to concentrate the samples. Protein concentration in the samples was measured using the DC Protein Assay Kit II (Bio-Rad Laboratories Inc).

For measurements of antioxidative activity, remodeled HDL species were separated by single-spin density gradient ultracentrifugation for 44 h according to Chapman *et al.* [6]

Non-denaturing polyacrylamide gradient gel electrophoresis (ND-PAGGE), western blotting and fluorescence imaging

The HDL particle size distribution was determined by non-denaturing gradient gel electrophoresis (ND-PAGGE) on 4–30% polyacrylamide gradient gels (LPE System, CBS Scientific Company Inc., Del Mar, CA). Molecular size markers were from GE Healthcare, (Amersham High Molecular Weight Calibration Kit for Electrophoresis, range: 17 nm thyroglobulin, 12.2 nm ferritin, 9.5 nm catalase, 8.4 nm lactate dehydrogenase, 7.1 nm BSA). Following separation by ND-PAGGE, proteins were transferred on to polyvinylidene difluoride membranes using the semi-dry blotting procedure (iBlot Gel Transfer Stacks, Invitrogen). For immunodetection of apoA-I, blots were probed with goat anti-human apoA-I polyclonal antibody (Rockland Immunochemicals Inc, # 600-101-109). ApoE was detected using apoE mAb (D6E10) [Abcam, ab 1906]. Fluorescence imaging of blots was performed using a Luminescent Image Analyzer (LAS4000, GE Healthcare) equipped with EPI-BGR light set 4000 (filters: Y515

BP Green LED and R670 Cy5 Red LED, to detect apoA-I labeled with DyLight 488 or DyLight 680 fluorophores, respectively).

Pull-down assay

Incubation mixtures (100 μL) containing biotinylated CSL112 or biotinylated HDL3 were diluted with PBS (1:1.5 v/v). Each diluted sample was divided into two equal aliquots. One aliquot was incubated with streptavidin sepharose and another one with sepharose 4B (both from GE Healthcare) with manual gentle mixing. To an aliquot of 120 μL, 50 μL of beads (50% slurry in PBS supplemented with 0.5% fatty acid free BSA) were added. After incubation for 15 min at room temperature, beads were removed by centrifugation.

Phospholipid analysis

Phospholipid contents were evaluated using an enzymatic assay (LabAssay, Wako Diagnostics). For the analysis of PC content of HDL samples by high performance liquid chromatography (HPLC), phospholipids were separated using a silica column (Ascentis Si 3μm, 2.1x150mm, Sigma-Aldrich). Isocratic elution was performed at a flow rate of 0.5 mL/min at 40°C. Elution buffer contained 87% (vol/vol) methanol and 13% (vol/vol) formate buffer (10 mM ammonium formate, 0.125% formic acid). Analytes were detected by a charged aerosol detector (Dionex Corona Ultra RS). A standard curve made with by soy L-α-phosphatidylcholine (Avanti Polar Lipids) was used for quantification.

Phospholipid and sphingolipid profiling of HDL samples was performed using an original liquid chromatography–mass spectrometry/mass spectrometry (LC/MS/MS) methodology as described by Camont *et al.*[7] In brief, HDL subfractions were pooled to provide total HDL (30 μg total phospholipid mass) and added to 4 mL of cold $CHCl_3$/acidified CH_3OH (5:2 v/v) containing seven internal lipid standards (Avanti Polar Lipids). A blank and quality controls (one every 5 samples) were extracted in parallel with each batch to ensure for quality control. K_4EDTA (200 mmol/L) solution was added (1:5 v/v) and the mixture was vortexed and centrifuged. The organic phase was dried under nitrogen and lipids were reconstituted into isopropanol/hexane/water (10:5:2 v/v), transferred into LC/MS vials, dried under nitrogen and resuspended in isopropanol/hexane/water (10:5:2 v/v). Eight principal phospholipid (PL) subclasses [Phosphatidylcholine (PC), lysophosphatidylcholine (LPC), phosphatidylethanolamine (PE), lysophosphatidylethanolamine (LPE), phosphatidylinositol (PI), phosphatidylglycerol (PG), phosphatidylserine (PS), and phosphatidic acid (PA)] and two principal sphingolipid (SL) subclasses [sphingomyelin (SM) and ceramide (Cer)] were assayed by LC/MS/MS. Lipids were quantified using a QTrap 4000 mass spectrometer (AB Sciex), an LC20AD HPLC system, and the Analyst 1.5 data acquisition system (AB Sciex). Quantification of PLs and SLs was performed in positive-ion mode, except for PI species that were detected in negative-ion mode. Sample (4 μL) was injected onto a Symmetry Shield RP8 3.5μm 2.1x50mm reverse phase column (Waters Corporation) using a gradient from 85:15 to 91:9 (v/v) methanol/water containing 5mmol/L ammonium formate and 0.1% formic acid at a flow rate of 0.1mL/min for 30 min. Lipid species were detected using multiple reaction monitoring reflecting the headgroup fragmentation of each lipid class and quantified using calibration curves specific for the ten individual lipid classes with up to 12 component fatty acid moieties; 17 calibration curves were generated in non-diluted and 10-fold diluted matrices to correct for matrix-induced ion suppression effects. More abundant lipid species which displayed a non-linear response in non-diluted extracts were quantified from a 10- or 100-fold diluted sample.

Electron microscopy

Negative stain electron microscopy (EM) was performed as described previously.[8] A glow-discharged thin carbon-coated 300-mesh copper grid was placed on a 100 µL drop of lipoprotein solution on Parafilm (0.005 mg/mL protein). After 1 min, the excess solution was removed by blotting with filter paper. The grid was washed by briefly touching the surface of the grid with a drop (200 µL) of water on Parafilm and then blotted dry with filter paper. The touching and blotting steps were repeated 3 times, each with a clean drop of water. The grid was placed on a drop (200 µL) of 1% (w/v) uranyl acetate solution for 1 min, then the excess solution was removed by blotting and the sample was air dried at room temperature. Specimens were examined with a FEI Tecnai F20 (FEI, Hillsboro, OR).

Cholesterol efflux assay

The capacity of the HDL to efflux cholesterol was assessed using [^{3}H]cholesterol-loaded RAW264.7 macrophages as previously described.[9] Briefly, RAW264.7 cells (mouse macrophages, ATCC) were cultured in DMEM (Dulbecco's modified Eagle's medium) supplemented with 10% (v/v) foetal calf serum (FCS), 2 mmol/L glutamine, 100 units/mL penicillin and 100 µg/mL streptomycin in a humidified 37 °C incubator in the presence of 5% CO_2.

On day 1, the cells used for efflux experiments were seeded into 24-well plates at a density of 0.35 x 10^6 cells per well, in 1 mL of medium. On day 2, cell culture medium was removed, and cells were labeled with cholesterol by incubation for 36 h in DMEM supplemented with 5% (v/v) FCS, 2 mmol/L glutamine and [1,2-^{3}H]cholesterol (1 µCi/mL; 0.5 µCi /0.5 mL of medium per well). Following the labeling period, cells were gently washed with PBS (on day 3) and then incubated in DMEM/2 mmol/L glutamine/0.2% fatty-acid-free BSA medium alone or stimulated with 0.3 mmol/L 8Br-cAMP (8 -bromoadenosine 3',5'-cyclic monophosphate sodium salt) for 16 h to up-regulate ABCA1. Efflux was promoted by incubating the [^{3}H]cholesterol- labeled RAW264.7 cells with each individual acceptor for 5 h. The difference in efflux between stimulated and non-stimulated cells is taken as a measure of ABCA1-mediated efflux.

Cholesterol efflux studies with BHK cells stably transfected with human ABCA1 cDNA or ABCG1 cDNA or SR-B1[10,11] were performed as described previously[12] with the following modifications. Cells were seeded into 24-well plates at a density of 0.15 x 10^6 cells per well, in 1 mL of DMEM containing 10% FCS. Next day, cells were labeled for 20 h with 1 Ci/ml of 3H-cholesterol in DMEM containing 10% FCS. Transporters were induced with 10 nM mifepristone in Dulbecco's modified Eagle's medium plus 0.2 mg/mL of fatty acid-free BSA for 18 h. Cholesterol efflux was measured after the addition of an acceptor in DMEM containing 10 nM mifepristone and 0.2 mg/mL of fatty acid-free BSA for 6 h. The difference in efflux between mifepristone-stimulated and non-stimulated cells is taken as a measure of transporter-mediated efflux.

Preparation of cellular extracts, detection of ATF3 by western blotting

To prepare cellular protein extracts, PBMC were washed three times in PBS and cellular proteins were precipitated with trichloroacetic acid at a final concentration 10%. Following centrifugation for 10 min, 14,000 rpm at 4°C, protein precipitates were washed twice in cold acetone, resuspended in electrophoresis sample buffer, separated on 4-12% pre-cast sodium dodecyl sulfate polyacrylamide gel electrophoresis (SDS-PAGE) gels (Novex,

Invitrogen) and blotted as described above. For immunodetection of ATF3, blots were probed with rabbit anti-ATF3 polyclonal antibody (sc-188, Santa Cruz Biotechnology).

Phospholipid hydroperoxide inactivating capacity of HDL

Reference LDL obtained from a healthy normolipidemic subject was preoxidized at 40 mg total cholesterol/dL with 4 mmol/L AAPH for 6 hours at 37°C. Oxidative modification was terminated by addition of EDTA (10 µmol/L) and BHT [10 µmol/L]. Oxidized LDL was dialyzed against PBS at 4°C to remove EDTA and excess BHT, and incubated at a concentration of 20 mg total cholesterol/dL for 4 hours in the presence or absence of HDL (8 mg total protein/dL) in PBS at 37°C. EDTA (100 µmol/L) was present to inhibit lipid peroxidation during the incubation. Phospholipid hydroperoxides were quantified in the reaction mixture before and after incubation by HPLC with chemiluminescent detection as described elsewhere.[13] To measure apoA-I content of native and oxidized methionine (Met) residues, HDL was subjected to HPLC with ultraviolet detection at 214 nm as previously described.[13]

Supplemental Figures

Supplementary Figure I. Addition of CSL112 to human pooled plasma *ex vivo* does not influence the size distribution of apoE-containing complexes. Human pooled cryo-depleted plasma (CSL Behring) was incubated alone or in the presence of CSL112 (1 mg protein/mL) for the indicated periods of time at 37°C. Samples of whole plasma or apoB-depleted plasma obtained after precipitation of apoB lipoproteins with polyethylene glycol were subjected to ND-PAGGE and analysed by western blotting with anti-apoA-I antibody (left panel) or with anti-apoE antibody (right panel). Sample of CSL112 (C) was included in the analysis as control.

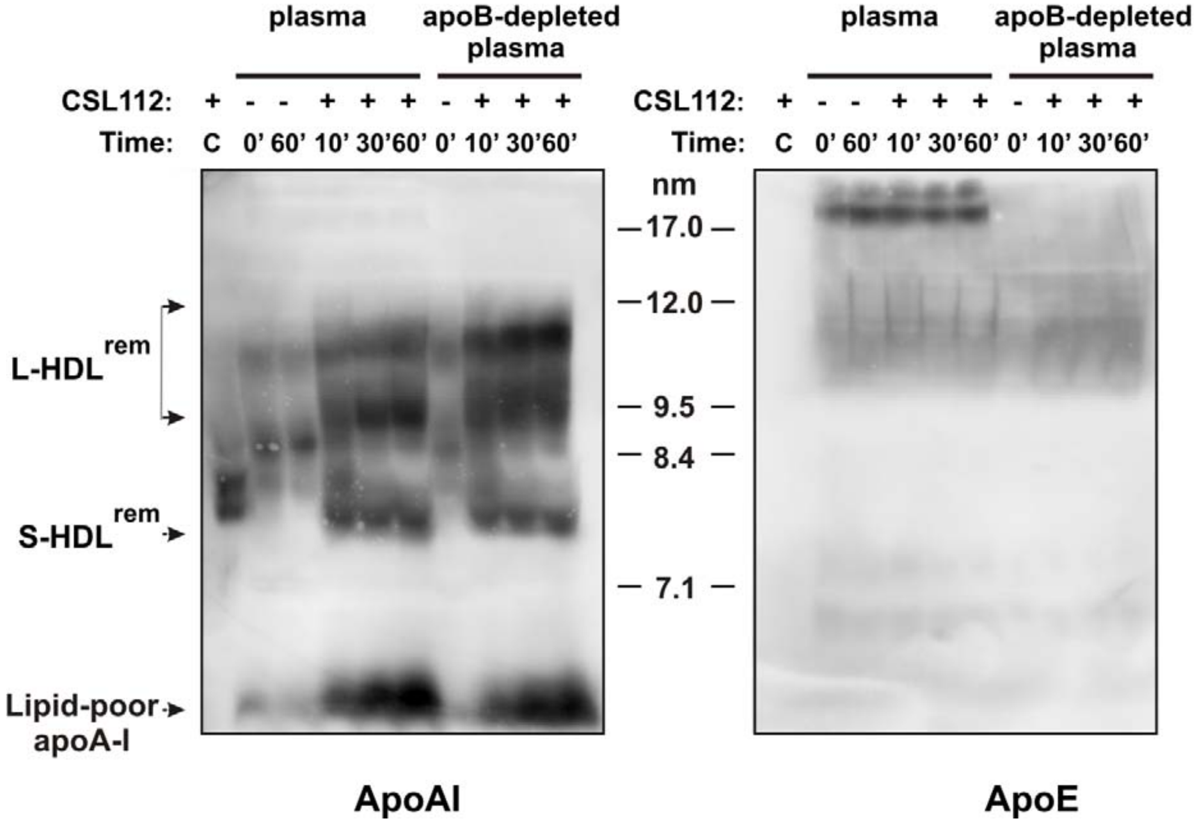

Supplementary Figure II. Apolipoprotein A-II concentrations following infusion of CSL112 in the Phase 1 single ascending dose study (Clinical Trial Registration: NCT1129661).[14, 15] (A) Unadjusted means (standard deviation [SD]) of cohorts as indicated (B) Baseline-corrected means (SD) of cohorts as indicated (C) Baseline-corrected means (SD) at t=2 hours (D) Baseline-corrected means (SD) at t=24 hours. Concentrations of apoA-II did not change immediately following the infusion of CSL112. However at 24 h following the infusion of CSL112 there was a modest dose-dependent increase in apoA-II not exceeding 10% of the baseline apoA-II concentration. This change could be explained by a slight decrease in apoA-II catabolism due to the overall increase of HDL.

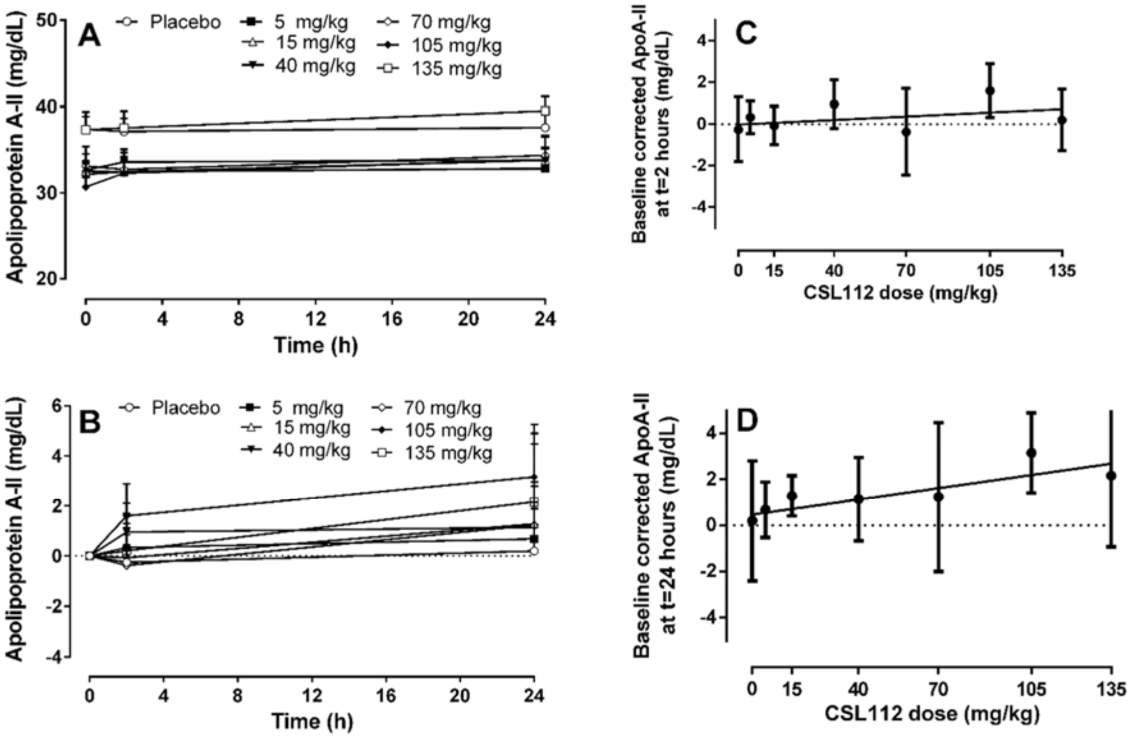

Supplementary Figure III. CSL112 promotes particle remodeling similarly *in vivo* and *in vitro.* In vivo: shown are plasma samples derived from healthy subjects infused over a 2 h period with CSL112 as part of completed phase I clinical trial NCT01281774[14, 15]. Donor 1, infusion dose of 75 mg/kg; Donor 2, infusion dose of 105 mg/kg; and Donor 3, infusion dose of 135 mg/kg. Blood was collected before the infusion (P) or at 2 and 8 h post infusion with CSL112. *In vitro*: CSL112 was incubated with normolipidemic human plasma at a final concentration of 1 mg protein/mL for the indicated periods of time at 37°C (Donor 4). Plasma samples were subjected to ND-PAGGE and analysed by western blotting with anti-apoA-I antibody. The positions of migration of HDL3, HDL2 subclasses and lipid-poor apoA-I are indicated.

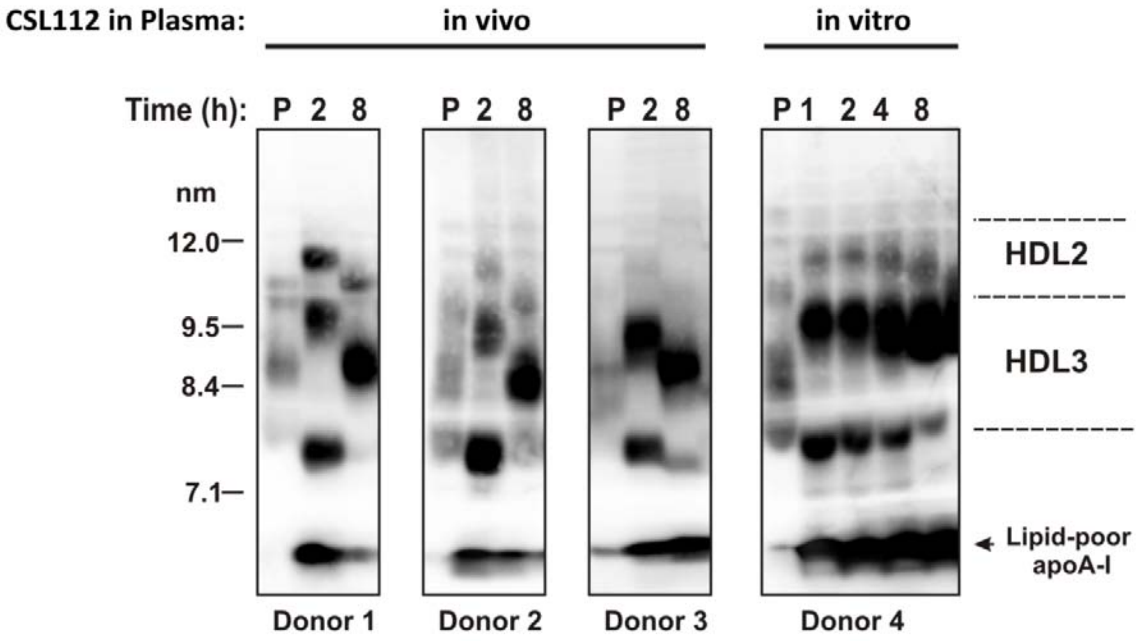

Supplementary Figure IV. Inhibition/stimulation of the plasma factors, CETP, LCAT, PLTP and sPLA2-IIA, does not affect the CSL112-induced remodeling of native HDL in human plasma *in vitro*.

Human fresh EDTA-plasma was preincubated at 4°C for 30 min in the presence or absence of either 10 µmol/L torcetrapib (CETP inhibitor), 2 mmol/L DTNB (LCAT inhibitor), 10 mmol/L 4-(2-aminoethyl)-benzenesulphonyl fluoride (AEBSF, a PLTP stimulator), or 100 µmol/L LY311727 (3-(3-acetamide-1-benzyl-2-ethyl-indolyl-5-oxy)propane phosphonic acid, a sPLA2-IIA inhibitor). CSL112 (final concentration of 1 mg protein/mL) was added to control plasma or plasma pretreated with individual inhibitors, and incubation continued at 37°C for 1 hour (A, C) or for the indicated time periods (B). **A,** Lipid-poor apoA-I levels were measured in plasma samples by a specific preβ1-HDL ELISA (Sekisui/ American Diagnostica GmbH). Mean concentrations ± standard deviation (SD) are derived from duplicate measurements. **B**, HDL fractions were subjected to non-denaturing gel electrophoresis on gradient 4-30% polyacrylamide gels followed by western blot analysis with an anti-apoA-I antibody. Data are shown for a representative experiment performed several times. **C,** HDL fractions were obtained after precipitation of plasma apolipoprotein B lipoproteins with polyethylene glycol[16] and used for the measurements of cholesterol efflux from RAW264.7 cells. Final concentration of CSL112 in efflux medium was 5 µg/mL; serum was at 0.45%. Each fractional cholesterol efflux value is the mean ± SD of triplicate measurements.

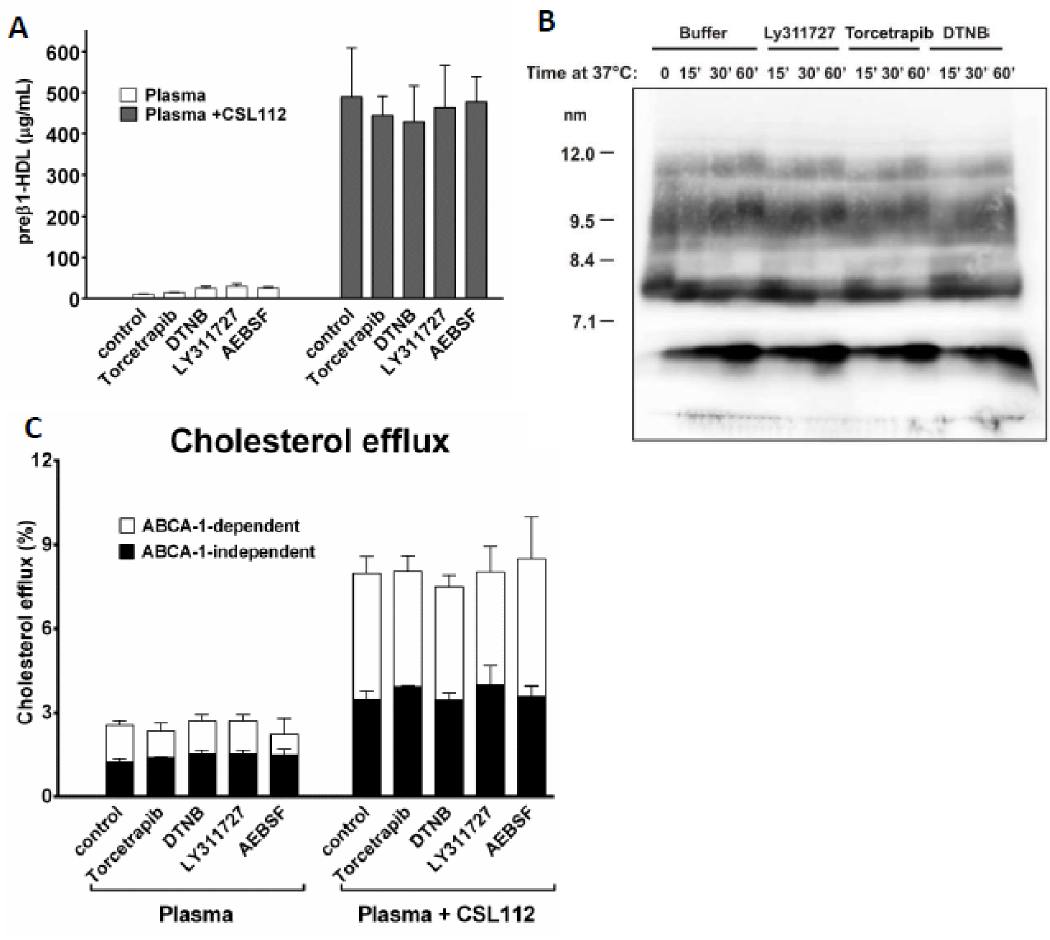

Supplementary Figure V. CSL112 causes the remodelling of endogenous HDL in plasma of wild-type, PLTP knockout or LCAT knockout mice *ex vivo.* Plasma samples of wild-type (WT), PLTP knockout (KO) and LCAT KO mice in a C57BL/6 background or normolipidemic human plasma (hPL) were incubated alone or in the presence of CSL112 for 1 h at 37°C. Final concentration of CSL112 was 0.75 mg protein/mL. Plasma samples were subjected to ND-PAGGE and analysed by western blotting with rabbit anti-mouse apoA-I antibody (Acris, # BP2052) (left panels). After stripping, membranes were reprobed with goat anti- human apoA-I antibody (Academy Bio-Medical Co, #11A-G2b) (right panels). **(A)** WT (n=2); PLTP KO (n=3). **(B)** WT (n=2); LCAT KO (n=3). Sample of CSL112 was included in the analysis as control (C). Plasma IDs are shown in square boxes below the lanes. The positions of migration of L-HDL^{rem}, S-HDL^{rem}, and lipid-poor apoA-I are indicated.

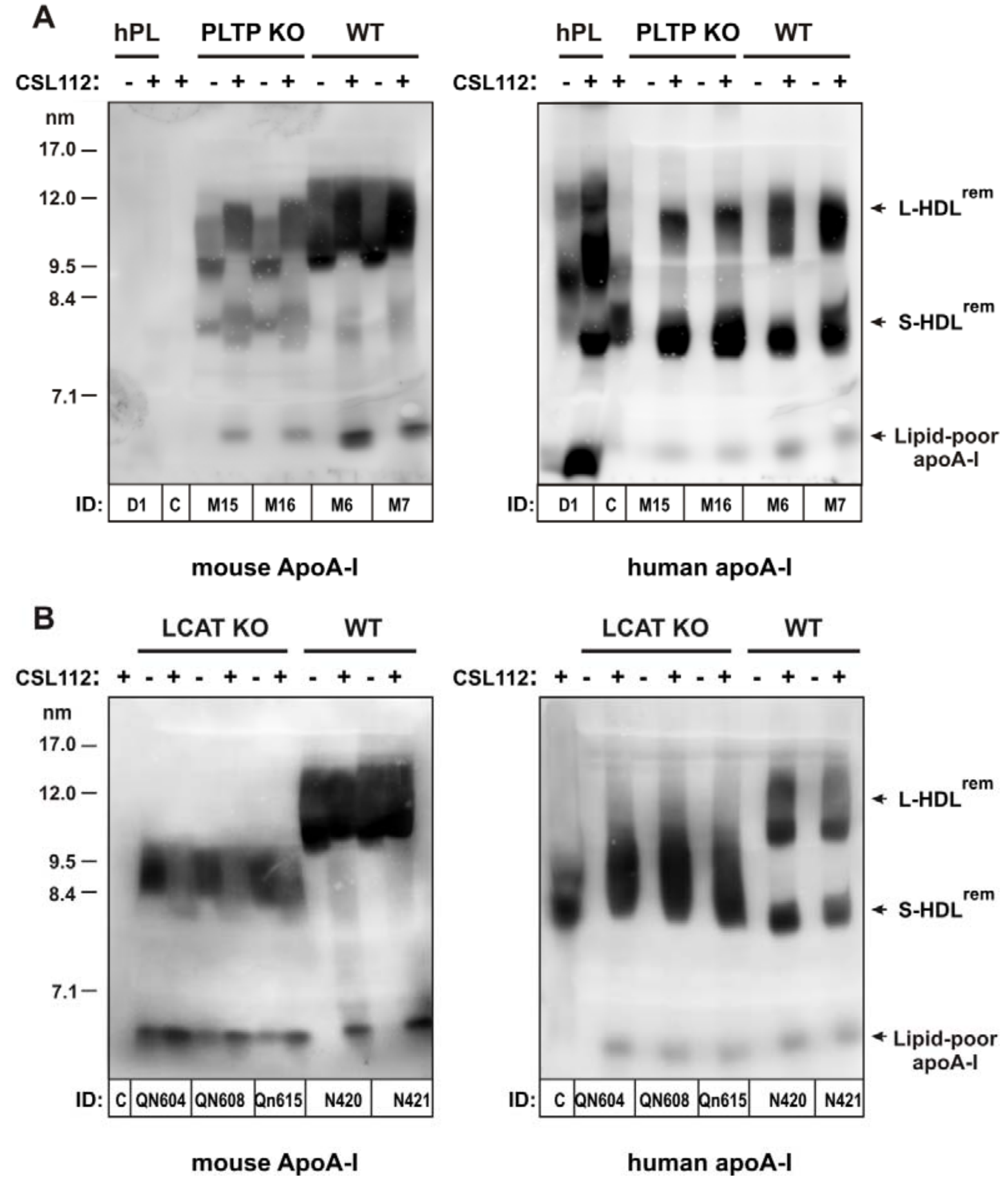

Supplementary Figure VI. Stability of parent lipoprotein preparations. CSL112, HDL3 or HDL2 were incubated in PBS for the indicated periods of time at 37°C at a final concentration of 1 mg protein/mL of each lipoprotein **(A)** Lipoproteins were subjected to ND-PAGGE and analysed by western blotting with anti-apoA-I antibody. **(B)** ABCA1-dependent and ABCA1-independent cholesterol efflux from RAW264.7 macrophages. Cells were loaded with free [^{3}H]cholesterol for 36 h, and stimulated with 0.3 mM 8Br-cAMP for 16 h to up-regulate ABCA1. Lipoproteins were used as cholesterol acceptors at a final protein concentration of 5 μg protein/mL in efflux medium, incubation was carried out for 5 h. Data of at least 3 independent experiments are shown. Values are presented as mean ± standard deviation.

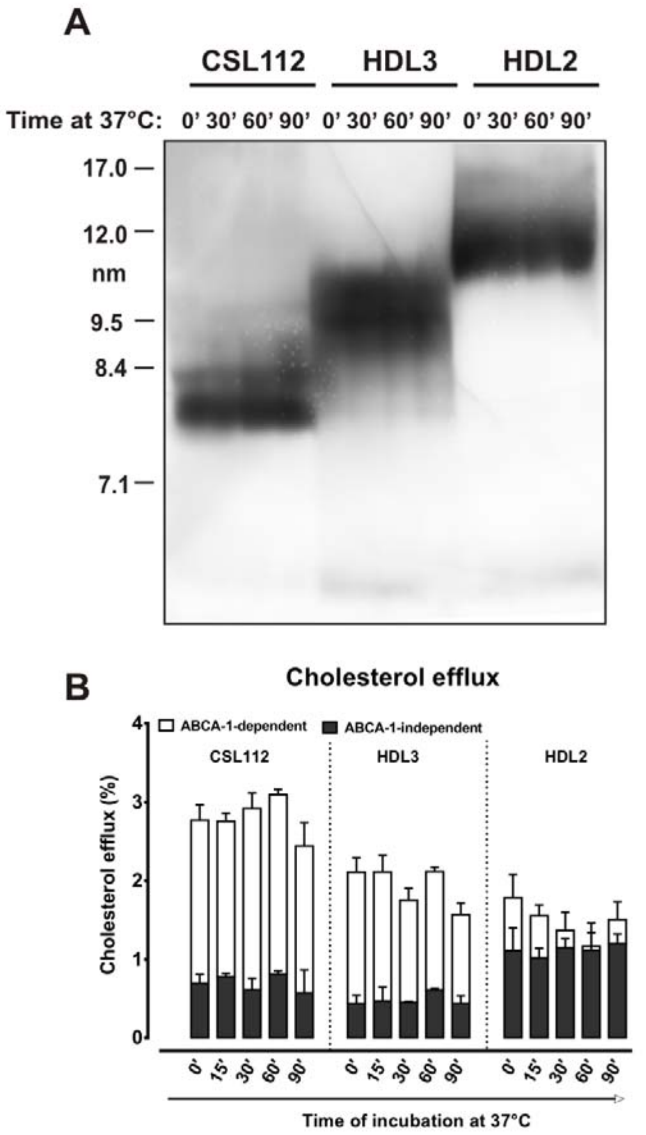

Supplementary Figure VII. Model for the interaction of CSL112 with HDL.

In the first step, CSL112 spontaneously fuses with plasma HDL generating a large unstable fusion product. The decay of this unstable fusion product may proceed along either of two paths. In path A, further remodeling leads to exchange of apoA-I between the particles and release of lipid-poor apoA-I. The resulting stable large HDL particles (L-HDL^{rem}) contain apoA-I and phospholipid from both parents while the lipid-poor apoA-I contains principally protein, also from both parents. In path B, further remodeling leads to the lipid exchange, but not to exchange of apoA-I before release of daughter particles. Rather, the parent HDL particle acquires only the lipid component from CSL112. The resulting remodeled large HDL (L-HDL^{rem}) contains apoA-I from HDL and additional phospholipid from CSL112 while small HDL (S-HDL^{rem}) particles contain apoA-I from CSL112 and a small amount of lipid, drawn from both parents.

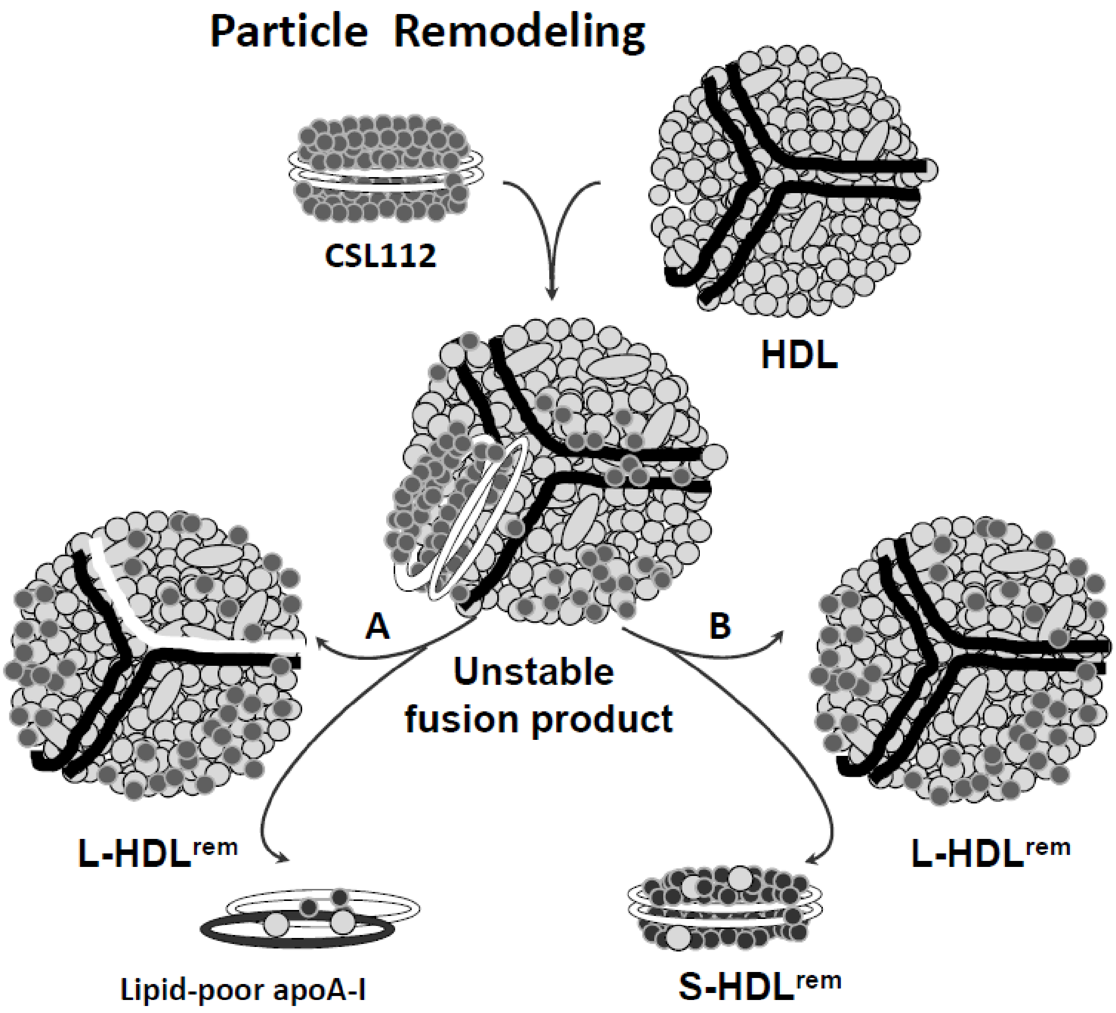

Supplementary Figure VIII. Analysis of isolated individual products of HDL remodeling by non-denaturating 1D gel electrophoresis.

CSL112 and HDL3 were incubated in PBS for 1 hour at 37°C, and products of the particle remodeling were isolated by sequential density gradient ultracentrifugation. Lipoproteins were separated by ND-PAGGE and visualized by Coomassie stain. Shown are isolated large HDL subspecies (Fr. 1; L-HDL^{rem}), small HDL subspecies (Fr. 2, S-HDL^{rem}), lipid poor apoA-I (Fr. 3). The positions of migration of remodeled HDL subspecies, L-HDL^{rem} and S-HDL^{rem}, and lipid poor apoA-I are indicated.

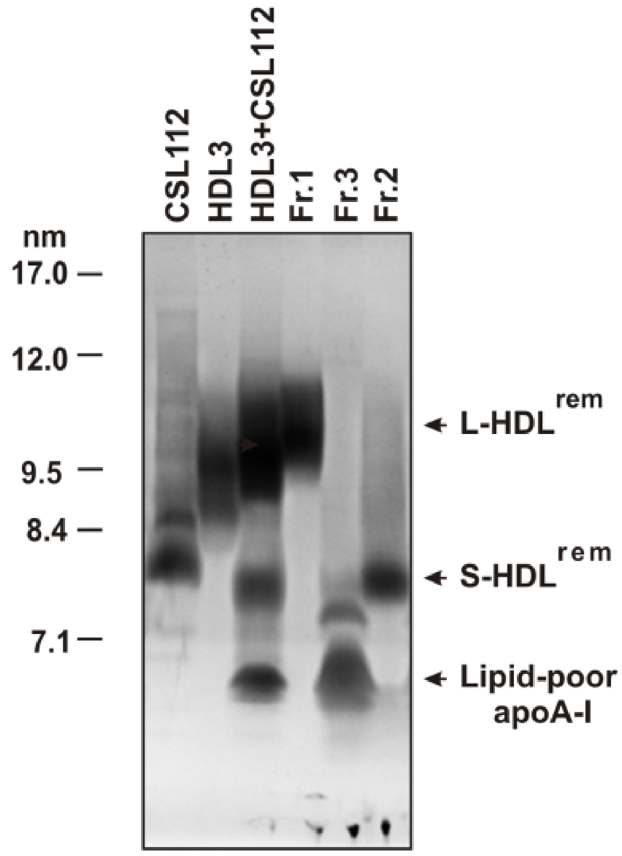

Supplementary Figure IX. Effects of the products of HDL remodeling on efflux cholesterol via ABCA1, ABCG1 or SR-B1 transporter. L-HDL^{rem}, S-HDL^{rem} and lipid-poor apoA-I, were purified from CSL112 and HDL3 incubation mixtures as described in Supplementary Figure VIII and used to efflux cholesterol from ABCA1-transfected (upper panel) or ABCG1-transfected (middle panel) or SR-B1-transfected BHK cells. Before the efflux, cells were labeled with free [^{3}H]cholesterol for 20 h, and then stimulated with 10 nM mifepristone for 18 h induce the transporters. Cholesterol acceptors were added on the basis of their protein content at a final concentration of 20 μg/mL and incubation was carried out for 6 h. Contribution of cholesterol efflux from the transporter was calculated as the difference in efflux between mifepristone-stimulated and non-stimulated cells. Each fractional efflux value represents the mean ± standard deviation for two independent experiments performed with different samples measured in triplicate. Phospholipid-poor acceptor particles, lipid-poor apoA-I and S-HDL^{rem}, were most efficient to efflux cholesterol from BHK cells expressing ABCA1. S-HDL^{rem} and L HDL^{rem} showed comparable activities to efflux cholesterol via ABCG1 which, unlike ABCA1, prefers phospholipid-rich acceptor particles. The most lipidated particle, L-HDL^{rem}, was found to mediate efflux via SR-B1 and by an aqueous diffusion process, which also requires phospholipid-rich acceptor particles.

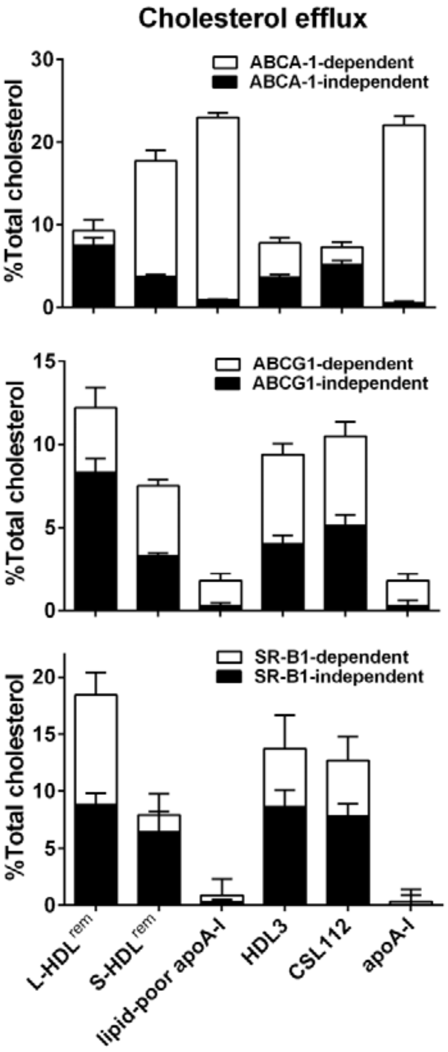

Supplementary Figure X. Similar effects of CSL112 on HDL remodeling and cholesterol efflux in whole blood and plasma *ex vivo*. CSL112 (1 mg protein/mL) was incubated with nonmolipidemic whole EDTA-blood (blood) or EDTA-plasma (plasma) for the indicated periods of time at 37°C. After the incubation, blood samples were briefly centrifuged to obtain cell-free supernatants. (**A)** Blood-derived and plasma samples were subjected to ND-PAGGE and analysed by western blotting with anti-apoA-I antibody. Samples of CSL112 and plasma alone (B) were included in the analysis as controls. **(B)** ApoB-depleted plasma was used for the measurements of ABCA1-dependent and ABCA1-independent cholesterol efflux from RAW264.7 cells. Final concentration of CSL112 in efflux medium was 5 µg/mL; plasma was at 0.45%. Each fractional cholesterol efflux value is the mean ± standard deviation of triplicate measurements. Data for one representative experiment out of three independent experiments are shown.

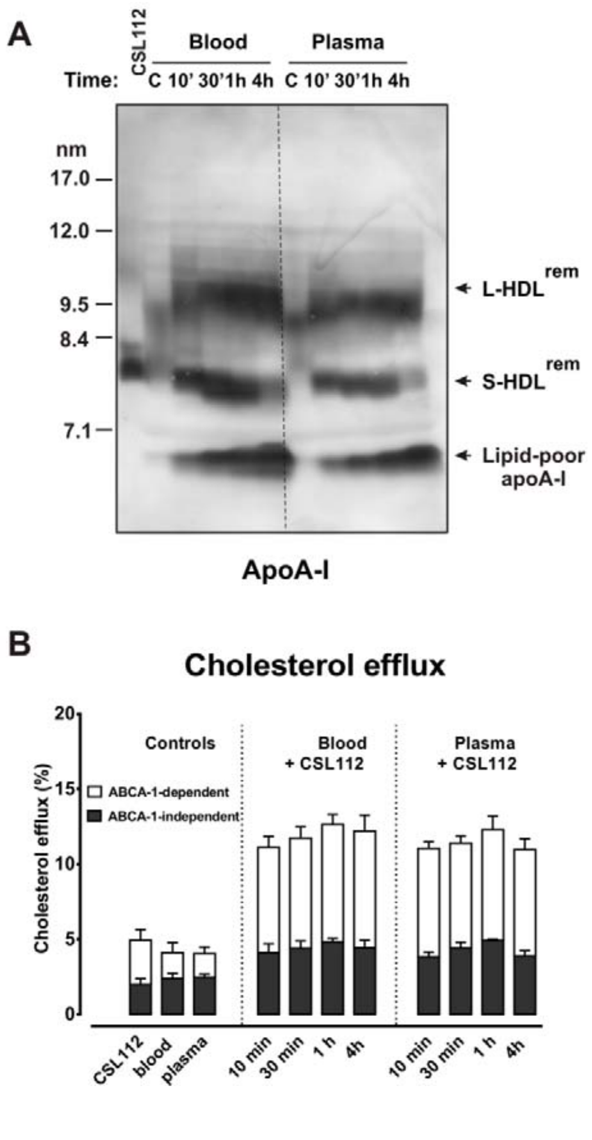

Supplementary Figure XI. Analysis of apoA-II distribution among the products of CSL112-induced remodeling of HDL3.

CSL112 was incubated with purified HDL3 for the indicated periods of time at 37°C. Lipoprotein mixtures were subjected to ND-PAGGE and analyzed by western blotting with an anti-apoA-II antibody (Abcam) (left panel) and an anti-apoA-I antibody (Rockland) (right panel). The position of migration of remodeled HDL subspecies, L-HDL^{rem} and S-HDL^{rem}, and lipid-poor apoA-I is indicated.

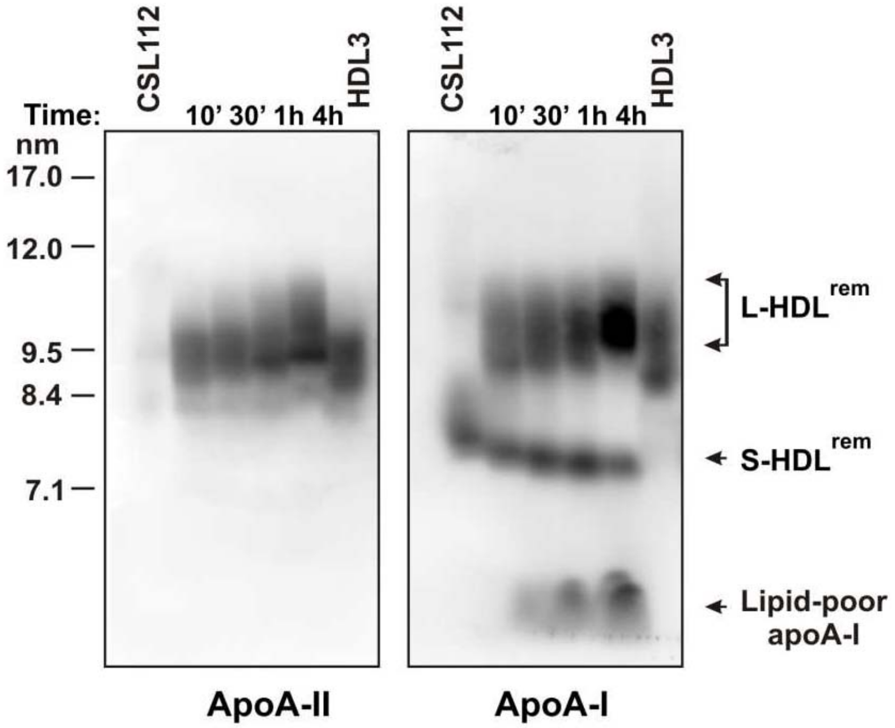

Supplementary Figure XII. Western blot analysis of apoE in CSL112 and purified HDL3 and HDL2 preparations. CSL112 and different samples of purified HDL2 (n=2) and HDL3 (n=3) were separated with 4-12% SDS-PAGE under reducing conditions. For lipoproteins, approximately 5 μg of total protein was loaded per lane. Amount of plasma protein loaded on a gel was equivalent to 2 μL of undiluted plasma (cryo-depleted plasma, CSL Behring). ApoE was detected using anti-human apoE antibody. After stripping, membranes were re-probed with anti-human apoA-I antibodies (right panel).

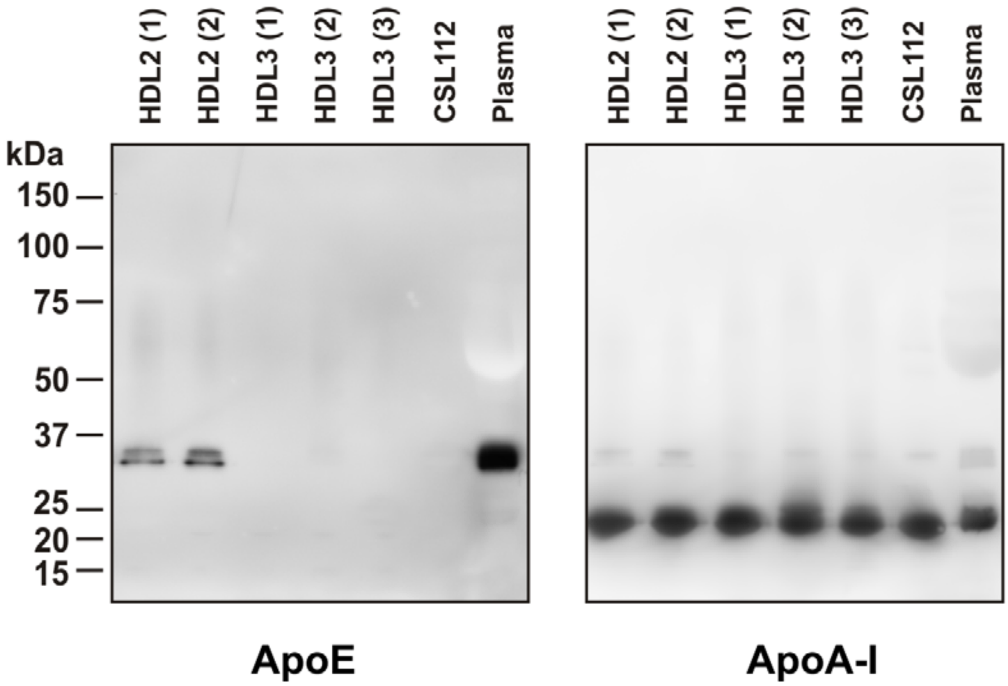

Supplemental Tables

Supplementary Table I. HDL particle content of choline-containing phospholipids

	**Ratio (Phospholipid : protein, mg/mg)**	
	HPLC*	***Enzymatic†***
**HDL3**	0.44 (0.42–0.45)	0.60 ± 0.1
**CSL112**	1.40 (1.33–1.37)	1.33 ± 0.08
**L-HDL^{rem}**	0.97 (0.92–1.02)	1.23 ± 0.18
**S-HDL^{rem}**	0.37 (0.29–0.45)	0.58 ± 0.11
**Lipid-poor apoA-I**	0.06 (0.05–0.07)	0.03 ± 0.03

* The mean (min–max) from two independent measurements performed with two different samples are shown.

† Data are shown as mean ± standard deviation (n≥3).

Supplementary Table II. Phospho- and sphingolipid composition of native, reconstituted and remodeled HDL

		HDL3 (n=2)	CSL112 (n=2)	L-HDL^{rem} (n=2)	S-HDL^{rem} (n=2)
**Mol of % PC**	**PC 18:2/18:2**	4.1 (3.1–5.2)	36.8 (36.2–37.5)	24.3 (23.5–25.2)	24.4 (22.9–25.9)
**Mol % of PL + SL**	**LysoPC**	2.8 (2.6–3.0)	1.9 (1.8–2.0)	4.06 (3.4–4.5)	3.78 (3.7–3.9)
	**LysoPE**	0.071 (0.066–0.076)	n.d.	0.030 (0.028–0.032)	0.069 (0.065–0.073)
	**PA**	0.051 (0.00–0.101)	0.015 (0.013–0.017)	0.045 (0.00–0.09)	0.067 (0.000–0.135)
	**PC**	73.9 (7.05–77.3)	98.1 (97.9–98.2)	84.7 (84.6–84.9)	84.7 (82.2–87.3)
	**PE**	2.5 (1.6–3.5)	n.d.	0.98 (0.92–1.04)	1.4 (1.3–1.5)
	**PG**	0.012 (0.008–0.016)	n.d.	0.008 (0.007–0.008)	0.012 (0.007–0.017)
	**PI**	5.3 (4.5–6.0)	n.d.	2.9 (2.6–3.1)	2.3 (2.1–2.5)
	**PS**	0.025 (0.013–0.037)	0.001 (0.001–0.001)	0.001 (0.000–0.002)	0.021 (0.000–0.042)
	**SM**	15.3 (12.4–18.2	n.d.	7.3 (7.1–7.7)	7.6 (5.1–10.0)
	**Ceramide**	0.035 (0.027-0.044)	n.d.	0.015 (0.012-0.019)	0.007 (0.005-0.009)

The mean (min–max) from two independent experiments performed with two different HDL samples are shown.

n.d., not detected; PA, phosphatidic acid; PC, phosphatidylcholine; PE, phosphatidylethanolamine; PG, phosphatidylglycerol; PI, phosphatidylinositol; PL, phospholipid; PS, phosphatidylserine; SL, sphingolipid; SM, Sphingomyelin

Supplementary References

1. Lund-Katz S, Nguyen D, Dhanasekaran P, Kono M, Nickel M, Saito H, Phillips MC. Surface plasmon resonance analysis of the mechanism of binding of apoA-I to high density lipoprotein particles. *J Lipid Res*. 2010;51:606-617
2. Martin-Nizard F, Richard B, Torpier G, Nouvelot A, Fruchart JC, Duthilleul P, Delbart C. Analysis of phospholipid transfer during HDL binding to platelets using a fluorescent analog of phosphatidylcholine. *Thromb Res*. 1987;46:811-825
3. Lambert G, Sakai N, Vaisman BL, et al. Analysis of glomerulosclerosis and atherosclerosis in lecithin cholesterol acyltransferase-deficient mice. *J Biol Chem*. 2001;276:15090-15098
4. Jiang XC, Qin S, Qiao C, Kawano K, Lin M, Skold A, Xiao X, Tall AR. Apolipoprotein B secretion and atherosclerosis are decreased in mice with phospholipid-transfer protein deficiency. *Nat Med*. 2001;7:847-852
5. Barter PJ, Chang LB, Newnham HH, Rye KA, Rajaram OV. The interaction of cholesteryl ester transfer protein and unesterified fatty acids promotes a reduction in the particle size of high-density lipoproteins. *Biochim Biophys Acta*. 1990;1045:81-89
6. Chapman MJ, Goldstein S, Lagrange D, Laplaud PM. A density gradient ultracentrifugal procedure for the isolation of the major lipoprotein classes from human serum. *J Lipid Res*. 1981;22:339-358
7. Camont L, Lhomme M, Rached F, Le Goff W, Negre-Salvayre A, Salvayre R, Calzada C, Lagarde M, Chapman MJ, Kontush A. Small, dense high-density lipoprotein-3 particles are enriched in negatively charged phospholipids: relevance to cellular cholesterol efflux, antioxidative, antithrombotic, anti-inflammatory, and antiapoptotic functionalities. *Arterioscler Thromb Vasc Biol*. 2013;33:2715-2723
8. Zhang L, Song J, Cavigiolio G, Ishida BY, Zhang S, Kane JP, Weisgraber KH, Oda MN, Rye KA, Pownall HJ, Ren G. Morphology and structure of lipoproteins revealed by an optimized negative-staining protocol of electron microscopy. *J Lipid Res*. 2011;52:175-184
9. Diditchenko S, Gille A, Pragst I, Stadler D, Waelchli M, Hamilton R, Leis A, Wright SD. Novel formulation of a reconstituted high-density lipoprotein (CSL112) dramatically enhances ABCA1-dependent cholesterol efflux. *Arterioscler Thromb Vasc Biol*. 2013;33:2202-2211
10. Vaughan AM, Oram JF. ABCA1 redistributes membrane cholesterol independent of apolipoprotein interactions. *J Lipid Res*. 2003;44:1373-1380
11. Sankaranarayanan S, Oram JF, Asztalos BF, Vaughan AM, Lund-Katz S, Adorni MP, Phillips MC, Rothblat GH. Effects of acceptor composition and mechanism of ABCG1-mediated cellular free cholesterol efflux. *J Lipid Res*. 2009;50:275-284
12. Remaley AT, Thomas F, Stonik JA, Demosky SJ, Bark SE, Neufeld EB, Bocharov AV, Vishnyakova TG, Patterson AP, Eggerman TL, Santamarina-Fojo S, Brewer HB. Synthetic amphipathic helical peptides promote lipid efflux from cells by an ABCA1-dependent and an ABCA1-independent pathway. *J Lipid Res*. 2003;44:828-836
13. Zerrad-Saadi A, Therond P, Chantepie S, Couturier M, Rye KA, Chapman MJ, Kontush A. HDL3-mediated inactivation of LDL-associated phospholipid hydroperoxides is determined by the redox status of apolipoprotein A-I and HDL particle surface lipid rigidity: relevance to inflammation and atherogenesis. *Arterioscler Thromb Vasc Biol*. 2009;29:2169-2175
14. Easton R, Gille A, D'Andrea D, Davis R, Wright SD, Shear C. A multiple ascending dose study of CSL112, an infused formulation of ApoA-I. *J Clin Pharmacol*. 2014;54:301-310

15. Gille A, Easton R, D'Andrea D, Wright SD, Shear CL. CSL112 enhances biomarkers of reverse cholesterol transport after single and multiple infusions in healthy subjects. *Arterioscler Thromb Vasc Biol*. 2014;34:2106-2114
16. Asztalos BF, de la Llera-Moya M, Dallal GE, Horvath KV, Schaefer EJ, Rothblat GH. Differential effects of HDL subpopulations on cellular ABCA1- and SR-BI-mediated cholesterol efflux. *J Lipid Res*. 2005;46:2246-2253
